# RNF149 modulates the type I IFN innate antiviral immune responses through degrading IRF3

**DOI:** 10.1371/journal.ppat.1013051

**Published:** 2025-04-17

**Authors:** Mengyun Wu, Jiamin Cai, Guodong Qiao, Xiaoping Li, Ji Zhou, Fei Xu, Yunfei Ye, Yufeng Wang, Xuena Xu, Jiaoyang Li, Xiaoyu Tian, Yu Shao, Chunsheng Dong, Zhengrong Chen, Chuangli Hao, Yi Yang, Jinping Zhang

**Affiliations:** 1 Institutes of Biology and Medical Sciences, Soochow University, Suzhou, China; 2 Department of Clinical Laboratory, The First Affiliated Hospital of Soochow University, Suzhou, China; 3 Department of Respiratory Medicine, Children’s Hospital of Soochow University, Suzhou, China; 4 The Fourth Affiliated Hospital, Institutes of Biology and Medical Science, SuZhou Medical College, Soochow University, Suzhou, China; Washington University School of Medicine in Saint Louis: Washington University in St Louis School of Medicine, UNITED STATES OF AMERICA

## Abstract

E3 ubiquitin ligases are key molecules in regulating the innate immune responses against virus. They catalyze the activation or degradation of various signaling proteins involved in the innate immune responses. Herein, we found the regulatory role of RNF149 in the host's innate immune responses against viral infection. Virus infection induced the expression of RNF149. Overexpression of RNF149 was associated with reduced production of IFN-β and enhanced viral replication. Mechanically, RNF149 interacted with IRF3 and downregulated its protein level. As an E3 ubiquitin ligase, RNF149 promoted the K27-linked ubiquitination of IRF3 at K409 and K33-linked ubiquitination at K366 and K409, which promoted IRF3 degradation through the proteasome pathway. Our results revealed the regulatory mechanism of RNF149 during viral infection and provided new insights into host cells responding to viral infection. Downregulating the expression of RNF149 may help enhance the antiviral ability of host cells and inhibit viral replication, thus providing a new strategy for the treatment of viral infection.

## Author Summary

E3 ubiquitin ligases play crucial roles in modulating innate immune responses to viral infection by regulating the activation or degradation of key signaling proteins. Type I interferons can induce the expression of interferon-stimulated genes and protect the body from viral invasion. Exploring the relationship between E3s and the type I interferon signaling pathway is of great significance for the treatment of viral infectious diseases. In this study, we identified RNF149 as a negative regulator in the host's immune response to viral infection. Upon viral infection, RNF149 expression was induced, facilitating viral replication possibly via suppressing the production of IFN-β. Mechanistically, RNF149 interacted with IRF3 and promoted K27-linked and K33-linked ubiquitination of IRF3, ultimately resulting in its degradation via the proteasome pathway. Our findings highlight the negative regulatory role of RNF149 in viral infection and provide new insights into how host cells respond to such infection.

## Introduction

In recent decades, Respiratory Syncytial Virus (RSV) has posed a significant burden on human health. Research indicates that Acute Lower Respiratory Tract Infections (ALRTI) are common among children, particularly those under 5 years old, and represent a major cause of mortality [1]. RSV is identified as the primary pathogen responsible for the infection. RSV infection is prevalent worldwide, with nearly all infants and toddlers under 2 years old having experienced RSV infection [2]. There is an urgent need for scientific approaches to prevent and treat RSV infection. Currently, there are no highly effective interventions against viral infection, underscoring the significance of exploring mechanisms for host resistance to RSV infection.

The innate immune response is the body's first line of defense against viral invasion. The recognition of viruses by the innate immune system relies on the binding of pattern-recognition receptors (PRRs) to pathogen-associated molecular patterns (PAMPs) [3]. The main pattern recognition receptors that can identify viral nucleic acids include Toll-like receptors (TLRs), Retinoic-acid-inducible gene I-like receptors (RLRs), Nucleotide-binding oligomerization domain-like receptors (NLRs), and cytoplasmic DNA sensors [4]. In the RLR signaling pathway, RIG-I and MDA5 can recruit the mitochondrial antiviral signaling protein (MAVS) and promote the production of Type I interferons through the IKKα/β-NF-κB and TBK1-IRF3/7 signaling pathways [5–7]. The classical Type I interferon signal transduction induces the expression of interferon-stimulated genes driven by ISRE (Interferon-Stimulated Response Element), many of which are found to promote a cellular antiviral state [8–11]. These interferon-stimulated genes can inhibit the viral invasion by suppressing viral transcription, translation, replication, nucleic acid degradation, or affecting cellular lipid metabolism processes [8,12].

Ubiquitination is a post-translational modification of proteins, and the ubiquitination of signal transduction proteins in the interferon signaling pathway is an essential regulatory mechanism of innate antiviral immunity [13]. In the innate immune response, most receptors and adapter proteins can be ubiquitinated and modified to transduce downstream signaling pathways, ultimately promoting the production of interferons and pro-inflammatory cytokines. For example, the K63-linked ubiquitination of RLRs, MAVS, and TBK1 is essential for their activation [14–18]. Besides, ubiquitination also negatively regulates signal transduction by promoting the degradation of signaling proteins [19]. For instance, the K48-linked ubiquitination of RLRs, MAVS and TBK1 leads to their degradation [20–22]. IRF3 is a key transcription factor in the Type I interferon signaling pathway, capable of regulating the production of Type I interferons [23]. Studies have shown that IRF3 is regulated by many E3 ubiquitin ligases, such as MID1, Ro52 and RAUL [24–26]. Currently, the research on the E3 ubiquitin ligases that regulate IRF3 and their mechanisms is not fully understood.

The E3 ubiquitin ligase RING finger protein 149 (RNF149) belongs to the RNF family, which contains a RING domain. It has been reported that RNF149 can bind to and ubiquitinate Serine/threonine-protein kinase B-raf (BRAF) for degradation [27]. Additionally, research has found that major facilitator superfamily domain containing 4 A (MFSD4A) can recruit RNF149 to degrade EPH receptor A2 (EPHA2), thereby inhibiting its downstream PI3K-AKT-ERK1/2 signaling pathway and epithelial-mesenchymal transition, thus inhibiting the proliferation and metastasis of nasopharyngeal carcinoma, providing new ideas and opportunities for targeted therapy of nasopharyngeal carcinoma [28]. In Alzheimer’s disease, the knockdown of RNF149 can ameliorate the symptoms of the disease [29]. Moreover, RNF149 is highly expressed in rat germ cells and plays a role in promoting their proliferation, but when the expression level of RNF149 decreases, the expression of germ cell differentiation-related genes increases [30]. The latest research shows that RNF149 can accelerate the progression of hepatocellular carcinoma by ubiquitinating the substrate DnaJ homolog subfamily C member 25 (DNAJC25) [31]. However, to date, the role of RNF149 in innate antiviral immune responses has not been reported.

Herein, we revealed the regulatory role of RNF149 in the host's innate immune responses against viral infection. RNF149 interacts with IRF3 and promotes its K27-linked and K33-linked polyubiquitination, which downregulates IRF3 protein level, suppresses the generation of IFN-β and diminishes the antiviral capabilities. In addition, Lys366 and Lys409 are the major residues of polyubiquitination induced by RNF149. The effects of RNF149 on viral infection depend on the E3 ubiquitin ligase activity. Therefore, RNF149 plays a vital role in innate antiviral immunity.

## Results

### 1. Viral infection upregulates the expression of RNF149

**Fig 1:**
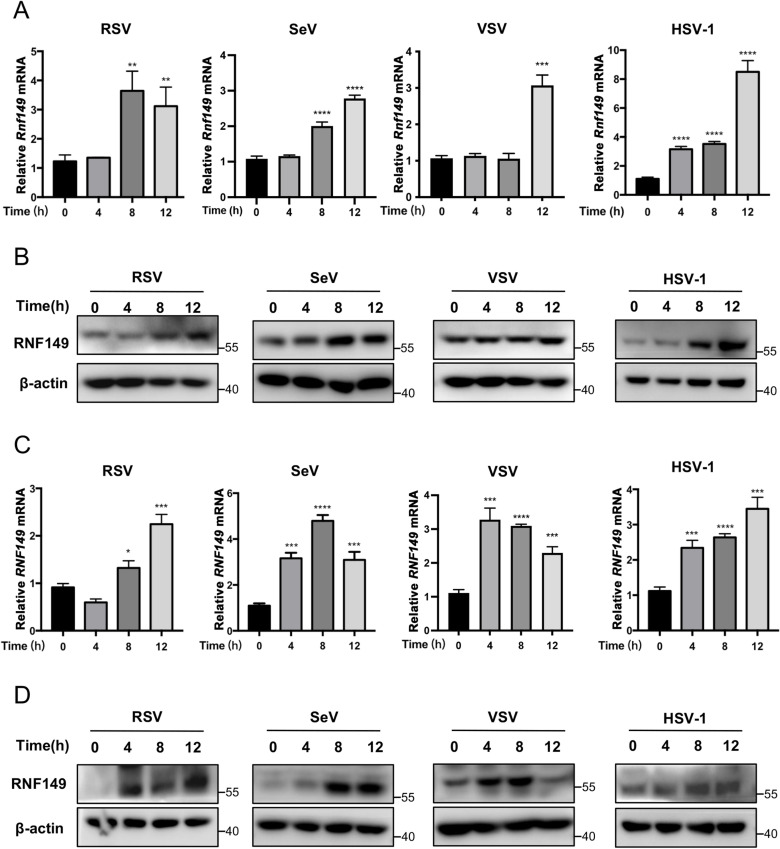
Both RNA and DNA virus infection induce RNF149 expression. (A) RT-qPCR analysis of RNF149 expression in RAW264.7 cells with the infection of RSV, SeV, VSV or HSV-1. n=3. Expression levels were normalized to 18S mRNA expression and then to the 0 h sample. (B) Western blot analysis of RNF149 expression in RAW264.7 cells with the infection of RSV, SeV, VSV or HSV-1. (C) RT-qPCR analysis of RNF149 expression in THP-1 cells with the infection of RSV, SeV, VSV or HSV-1. n=3. Expression levels were normalized to 18S mRNA expression and then to the 0 h sample. (D) Western blot analysis of RNF149 expression in THP-1 cells with the infection of RSV, SeV, VSV or HSV-1. The *P*-value was determined using an unpaired *t*-test. *P < 0.05, **P < 0.01, ***P < 0.001, ****P < 0.0001. Data are representative of three independent experiments.

E3 ubiquitin ligases play a crucial regulatory role in the innate immune response against viral infection. To further explore key E3 ubiquitin ligases that play important roles in viral infection, we established a virus-infected macrophage model. Previously, we infected mouse macrophage cell line RAW264.7 cells with RSV type L19 tagged with mCherry and used protein mass spectrometry to examine the protein expression profiles of RAW264.7 cells with or without viral infection. The E3 ubiquitin ligases were identified and listed in Table 1 through analysis. Notably, the expression of E3 ubiquitin ligase RNF149 showed the most significant upregulation after RSV infection. To verify the result, we infected RAW264.7 cells with RSV and detected the expression of RNF149 by RT-qPCR and Western blot assay. The results showed that RSV infection upregulated the expression of RNF149 in both mRNA and protein levels (Fig 1A–B). To confirm whether this phenomenon is RSV-specific, we infected RAW264.7 cells with RNA viruses SeV, VSV and DNA virus HSV-1, respectively. The RT-qPCR and Western blot results showed that the expression of RNF149 in macrophages increased after virus infection (Fig 1A–B). Moreover, in the human monocytic leukemia THP-1 cells, both RNA virus and DNA virus infection could also upregulate the expression of RNF149 in the mRNA and protein levels (Fig 1C–D). These results suggest that RNA and DNA virus infection can upregulate the expression of RNF149 in macrophages.

**Table 1:** The expression of E3 ubiquitin ligase before and after RSV infection.

Name	Abundance Ratio: (rsv1)/ (nc1)	Abundance Ratio: (rsv2)/ (nc2)	Abundance Ratio: (rsv3)/ (nc3)
Rnf149	8.328	3.412	3.137
Rnf213	3.386	1.112	1.922
Dtx3l	2.422	1.016	2.478
Rnf114	1.786	0.967	1.52
Ppp1r11	1.537	1.552	1.462
Trim32	1.488	0.787	1
Rbbp6	1.436	0.596	1.118
Ltn1	1.293	1.306	0.702
Trim56	1.254	1.018	1.162
Uhrf2	1.173	0.01	1.003
Trip12	1.165	1.099	1.26
Ppil2	1.095	1.095	0.838
Hectd4	1.086	0.934	0.646
Ube3c	1.073	0.898	0.936
Hectd1	1.058	1.246	0.867
Herc4	1.038	0.811	0.835
Rbx1	1.032	1.026	0.75
Maea	1.027	0.01	0.01
Ube2o	0.975	0.894	0.803
Ubr1	0.954	1.167	1.036
Ubr4	0.919	0.792	0.878
Ufl1	0.904	0.679	0.891
Rnf20	0.903	1.215	0.807
Ubr5	0.886	1.36	0.835
Ranbp2	0.843	0.888	1.208
Rnf25	0.835	0.394	1.013
Jade2	0.802	0.394	1.302
Cbl	0.795	1.261	1.029
Arih1	0.758	1.035	0.664
Rnf40	0.709	0.709	0.663
Ube2e3	0.679	0.849	0.916
Trim33	0.637	1.377	1.274
Rnf2	0.633	0.814	1.014
Uhrf1	0.438	1.489	0.765
Znrf2	0.01	0.01	1.726
Rad18	0.01	0.765	0.01

Upon viral infection, Type I interferons are produced to combat the virus. Type I interferons bind to their receptor IFNAR, which subsequently activates the transcription factors STAT1 and STAT2 and leads to the transcription of a multitude of interferon-stimulated genes [32]. To explore whether the upregulation of RNF149 expression following viral infection is regulated by the downstream signaling pathway of Type I interferon, we stimulated RAW264.7 cells with a gradient of different concentrations of IFN-β. The results of RT-qPCR and Western blot showed that the expression level of RNF149 significantly increased after stimulation with IFN-β (S1A–B Fig). Furthermore, the Type I interferon receptor was blocked with an IFNAR1 antibody, followed by infection with RSV in RAW264.7 cells. The RT-qPCR results showed that the upregulation of RNF149 expression induced by viral infection was suppressed in the IFNAR1 antibody-blocking group (S1C Fig).

The CHIP-Seq results recorded on the ENCODE website indicate that STAT1 can bind to the promoter region of RNF149 in HeLa cells (S1D Fig). To confirm whether STAT1 is involved in regulating RNF149 expression, we conducted a dual-luciferase reporter gene assay. Overexpression of STAT1 increased the RNF149 promoter activity (S1E Fig). Although STAT2 cannot directly bind to DNA, it can effectively promote transcriptional activation [33]. Therefore, we transfected RAW264.7 cells with STAT1 and STAT2 specific siRNAs, followed by infection with RSV. The RT-qPCR and Western blot results showed that the knockdown of STAT1 and STAT2 significantly inhibited the upregulation of RNF149 expression induced by RSV in macrophages (S1F–G Fig). These results suggest that the expression of RNF149 in macrophages induced by viral infection is regulated by the downstream signaling pathway of Type I interferon.

### 2. RNF149 promotes viral replication probably by downregulating IFN-β production

**Fig 2:**
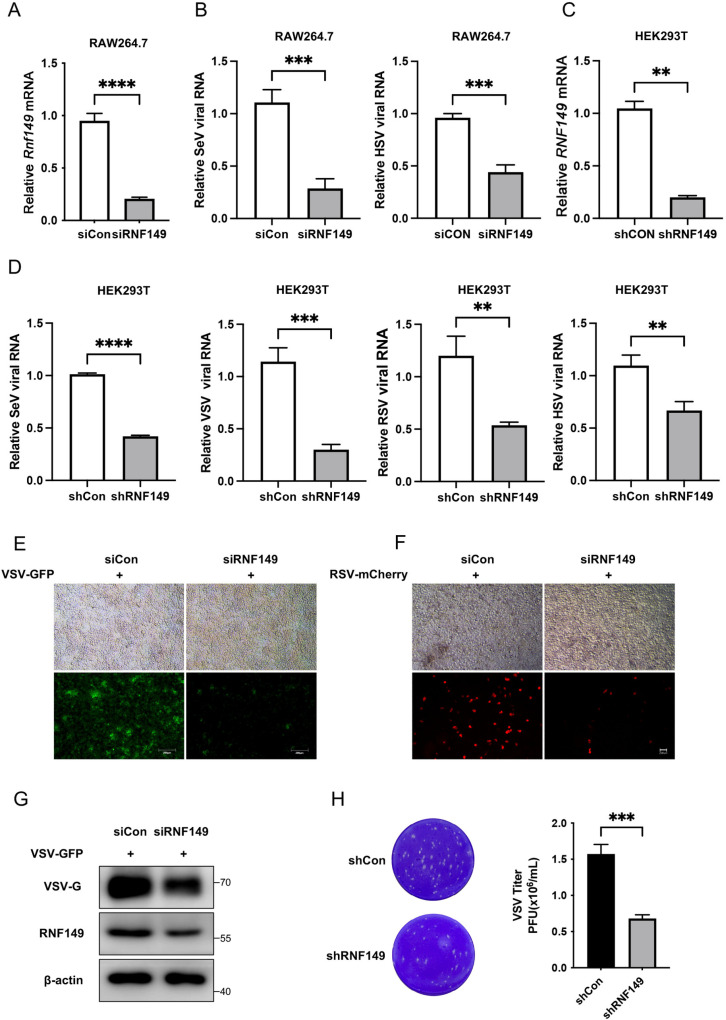
Knocking down of RNF149 reduces viral replication. (A-B) RAW264.7 cells transfected with control siRNA or siRNF149 for 36 h were infected with SeV or HSV-1 for 12 h, and the expression of *Rnf149* and virus RNA was detected by RT-qPCR. n=3. Expression levels were normalized to 18S mRNA expression and then to the siCon sample. (C-D) HEK293T cells transfected with shCon or shRNF149 for 36 h were infected with SeV, VSV, RSV or HSV-1 for 12 h, and the expression of RNF149 and virus RNA was detected by RT-qPCR. n=3. Expression levels were normalized to 18S mRNA expression and then to the shCon sample. (E) HEK293T cells transfected with siCon or siRNF149 for 36 h were infected with VSV-GFP for 12 h, and the fluorescence of VSV was detected by fluorescence microscopy. Scale bar, 200 μm. (F) HEK293T cells transfected with siCon or siRNF149 for 36 h were infected with RSV-mCherry for 12 h. The fluorescence of RSV was detected by fluorescence microscopy. Scale bar, 200 μm. (G) HEK293T cells transfected with siCon or siRNF149 for 36 h were infected with VSV-GFP for 12 h, and the expression of VSV-G was detected by Western blot. (H) HEK293T cells were transfected with shCon or shRNF149 for 36 h and infected with VSV-GFP for 12 h. The VSV viral load was detected by plaque assay. n=3. The *P*-value was determined using an unpaired *t*-test. **P < 0.01, ***P < 0.001, ****P < 0.0001. Data are representative of three independent experiments.

To further explore the impact of RNF149 on the innate antiviral response, we designed siRNA targeting mouse *Rnf149* and transfected it into RAW264.7 cells. The mRNA level of *Rnf149* decreased after transfection with RNF149 siRNA (Fig 2A). Knocking down of RNF149 reduced the replication of SeV and HSV in RAW264.7 cells (Fig 2B). To further verify this phenomenon, we knocked down RNF149 in HEK293T cells by transfecting with shRNF149 plasmid. The results demonstrated that knocking down RNF149 also suppressed the replication of SeV, VSV, RSV and HSV in HEK293T cells (Fig 2C–D). Moreover, an immunofluorescence assay was carried out in HEK293T cells infected with VSV-GFP or RSV-mCherry. The results demonstrated that knocking down of RNF149 reduced viral infection, as shown by decreased GFP and mCherry signals (Fig 2E–F). Additionally, we confirmed the effect of RNF149 knockdown on virus infection by Western blot and plaque assay (Fig 2G–H). We also verified the impact of RNF149 on virus infection by overexpression in HEK293T cells. RNF149 overexpression promoted the viral replication of SeV, VSV, RSV and HSV (S2A Fig). The immunofluorescence assay showed that overexpression of RNF149 promoted viral infection, as shown by increased GFP and mCherry signals (S2B–C Fig). Western blot and plaque assay both confirmed the function of RNF149 overexpression on viral infection (S2D–E Fig).

**Fig 3:**
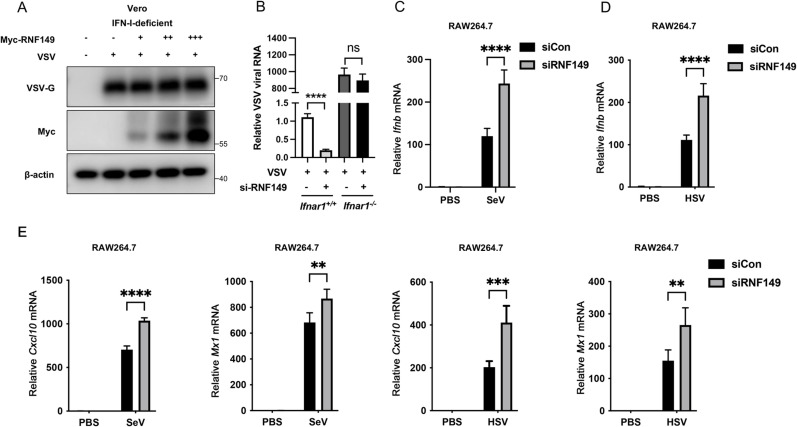
The regulation of RNF149 on virus replication is associated with IFN-I signaling. (A) Western blot analysis of VSV-G protein level in Vero cells transfected with an increased amount of Myc-RNF149 for 36 h and infected with VSV-GFP for 12 h. (B) The expression of VSV RNA in *Ifnar1*^*+/+*^ and *Ifnar1*^*−/−*^ MEF cells transfected with siCon or siRNF149 and infected with VSV for 12 h was detected by RT-qPCR. Expression levels were normalized to 18S mRNA expression and then to the *Ifnar1*^*+/+*^-siRNF149 sample. (C-D) The expression of *Ifnb* mRNA in RAW264.7 cells transfected with control siRNA or siRNF149 for 36 h and infected with SeV or HSV-1 for 12 h was detected by RT-qPCR. n=3. Expression levels were normalized to 18S mRNA expression and then to the si-Con-PBS sample. (E) RT-qPCR analysis of *Cxcl10* and *Mx1* mRNA in RAW264.7 cells transfected with control siRNA or siRNF149 for 36 h and infected with SeV or HSV-1 for 12 h. n=3. Expression levels were normalized to 18S mRNA expression and then to the si-Con-PBS sample. (B) The *P*-value was determined using an unpaired *t*-test. (C-E) The *P*-value was determined using a two-way ANOVA test. **P < 0.01, ***P < 0.001, ****P < 0.0001, ns, not significant. Data are representative of three independent experiments.

Upon viral infection, the body activates the innate immune response to combat the virus through mechanisms such as the interferon signaling pathway. Type I interferon (IFN-I) responds early during viral infection and can induce the expression of many interferon-stimulated genes. Therefore, we next investigated whether RNF149 negatively regulates the innate antiviral responses by affecting the IFN-I signaling pathway. Firstly, we overexpressed RNF149 in IFN-I-deficient Vero cells and infected them with VSV. Western blot analysis showed that RNF149 overexpression did not alter the VSV-G protein levels in Vero cells (Fig 3A). Besides, we used *Ifnar1*^*+/+*^ and *Ifnar1*^*−/−*^ MEF cells. MEF cells were transfected with siCon or siRNF149 and then infected with VSV. The results showed that knockdown of RNF149 reduced viral infection in *Ifnar1*^*+/+*^ cells. However, the knockdown of RNF149 did not affect viral infection in *Ifnar1*^*−/−*^ MEF cells (Fig 3B). It’s indicated that the regulation of viral infection by RNF149 is associated with IFN-I signaling. Type I interferon primarily consists of IFN-α and IFN-β. IFN-β is the first cytokine to respond during viral infection and can be produced by various immune cells. In contrast, IFN-α expression is relatively delayed and mainly originates from plasmacytoid dendritic cells. Therefore, we focused on detecting IFN-β. RNF149 knockdown in RAW264.7 cells increased the production of *Ifnb* with the infection of both SeV and HSV-1 (Fig 3C–D). Besides, we examined the expression of ISGs *Cxcl10* and *Mx1*. The results showed that, after RNF149 knockdown, cells produced higher levels of *Cxcl10* and *Mx1* following viral infection (Fig 3E). In addition, we verified the results in HEK293T cells. The results showed that overexpression of RNF149 led to lower levels of IFN-β mRNA compared to the control group with virus infection (S3A Fig). Conversely, RNF149 knockdown promoted the IFN-β expression after virus infection (S3B Fig). In line with the increased IFN-β, RNF149 knockdown upregulated the expression of interferon-stimulated genes (ISGs) *IFIT1*, *ISG15*, and *ISG54* after SeV infection (S3C Fig). Taken together, we demonstrate that RNF149 limits IFN-β production during viral infection and negatively regulates innate antiviral responses.

### 3. RNF149 deficiency protects mice against viral infection

To further investigate the regulatory role of RNF149 on viral infection, *Rnf149*^*−/−*^ mice were constructed using CRISPR-Cas9 technology, and the exons 2 to 5 of *Rnf149* were deleted (S4A Fig). *Rnf149*^*−/−*^ mice were viable and normal in size. DNA sequencing of the mouse genome and PCR analysis of mouse tail DNA also confirmed the successful construction of *Rnf149*^*−/−*^ mice (S4A–B Fig). Additionally, RT-qPCR and Western blot analysis indicated that *Rnf149* was successfully knocked out in macrophages (S4C–D Fig).

**Fig 4:**
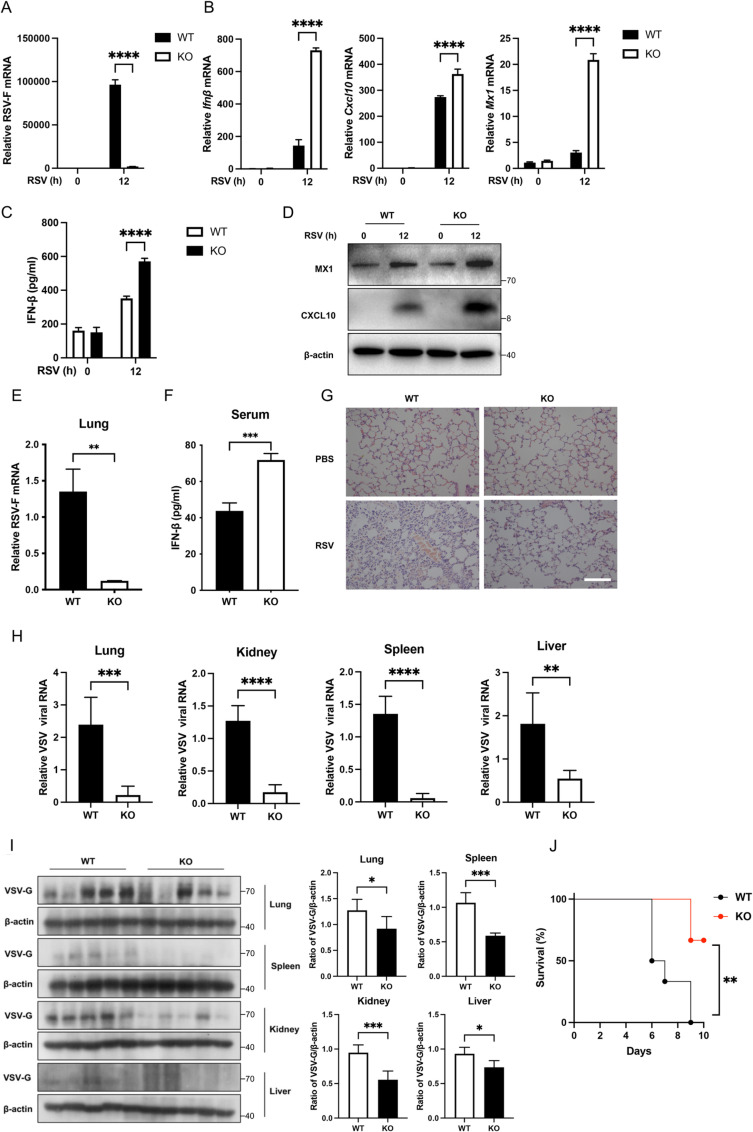
RNF149 deficiency protects mice against viral infection. (A) The expression of RSV-F mRNA in wild-type (WT) and *Rnf149*^*−/−*^ (KO) mouse peritoneal macrophages infected with RSV. n=3. Expression levels were normalized to 18S mRNA expression and then to the WT-0 sample. (B) The expression of *Ifnb*, *Cxcl10* and *Mx1* mRNA in WT and KO mouse peritoneal macrophages infected with RSV. n=3. Expression levels were normalized to 18S mRNA expression and then to the WT-0 sample. (C) ELISA of IFN-β expression in supernatant from WT and KO peritoneal macrophages infected with RSV. n=3. (D) The protein level of Cxcl10 and Mx1 in WT and KO mouse peritoneal macrophages infected with RSV. (E) RT-qPCR analysis of RSV expression in the lungs of WT and KO mice infected with RSV (1×10^9^ PFU/mouse) for 3 days through nasal intubation drip. n=3. Expression levels were normalized to 18S mRNA expression and then to the WT-0 sample. (F) ELISA of IFN-β expression in serum from WT and KO mice infected with RSV (1×10^9^ PFU/mouse) for 3 days through nasal intubation drip. n=3. (G) HE staining of lungs from WT and KO mice infected with RSV (1×10^9^ PFU/mouse) for 3 days through nasal intubation drip. Scale bar, 100 μm. (H) RT-qPCR analysis of VSV expression in the lungs, kidneys, spleens and livers from WT and KO mice infected with VSV (4×10^8^ PFU/mouse) for 24 h through intraperitoneal injection. n=5. Expression levels were normalized to 18S mRNA expression and then to the WT sample. (I) Western blot analysis of VSV-G expression in the lungs, spleens, kidneys and livers from WT and KO mice infected with VSV (4×10^8^ PFU/mouse) for 24 h through intraperitoneal injection, and quantification of band intensity of VSV-G in the blot, presented relative to β-actin. n=5. (J) Survival of WT and KO mice given intraperitoneal injection of VSV (4×10^8^ PFU/mouse). n=6. (A-C) The *P*-value was determined using a two-way ANOVA test. (E, F, H, I) The *P*-value was determined using an unpaired *t*-test. (J) The *P*-value was determined using a log-rank (Mantel-Cox) test. *P < 0.05, **P < 0.01, ***P < 0.001, ****P < 0.0001. Data are representative of three independent experiments.

Next, we further verified the regulation of RNF149 on viral infection in primary mouse peritoneal macrophages. We infected the peritoneal macrophages from WT and KO mice with RSV. Compared to the control group, the expression of RSV-F mRNA was inhibited in *Rnf149*^*−/−*^ macrophages (Fig 4A). Meanwhile, the mRNA level of *Ifnb* and its ISGs *Cxcl10* and *Mx1* was upregulated in *Rnf149*^*−/−*^ macrophages infected with RSV relative to these genes in WT macrophages (Fig 4B). Besides, the protein level of Ifnb, Cxcl10 and Mx1 was higher in *Rnf149*^*−/−*^ macrophages infected with RSV than in WT macrophages (Fig 4C–D). To further elucidate the function of RNF149 in innate antiviral immunity in vivo, we challenged WT and KO mice with RSV via nasal instillation. The expression of RSV-F mRNA in the lungs of KO mice was lower than that in the WT group (Fig 4E). ELISA analysis showed that IFN-β in *Rnf149*^*−/−*^ serum was higher than that in the WT group (Fig 4F). H&E staining of lungs after RSV infection showed less injury in *Rnf149*^*−/−*^ mice than in WT mice (Fig 4G). Moreover, we also challenged WT and KO mice with VSV through intraperitoneal injection. RT-qPCR and Western blot results showed that compared to the WT mice, the VSV load in the lungs, kidneys, spleens and livers of the *Rnf149*^*−/−*^ mice was significantly reduced (Fig 4H–I). Furthermore, *Rnf149*^*−/−*^ mice exhibited a higher survival rate compared to the WT mice, demonstrating a stronger resistance to VSV infection (Fig 4J). Collectively, these results suggest that RNF149 deficiency enhanced innate antiviral immunity.

### 4. RNF149 regulates viral replication through IRF3

**Fig 5:**
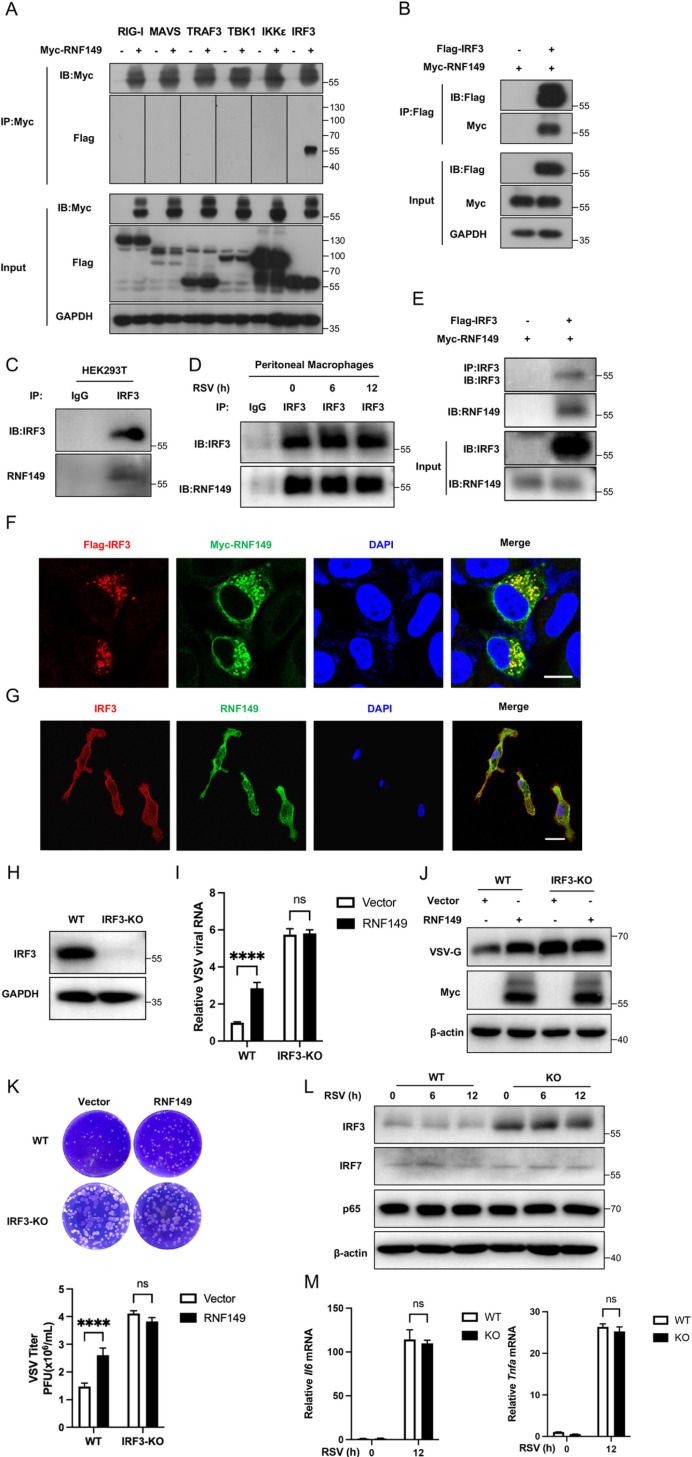
RNF149 regulates viral replication through IRF3. (A) Co-IP analysis of Myc-RNF149 along with Flag-RIG-I, Flag-MAVS, Flag-TRAF3, Flag-TBK1, Flag-IKKε and Flag-IRF3 plasmids in HEK293T cells using an anti-Myc antibody. (B) HEK293T cells were transfected with Myc-RNF149 and Flag-IRF3 plasmids, and their interaction was detected by Co-IP through an anti-Flag antibody. (C) Endogenous interaction of RNF149 and IRF3 was detected by Co-IP in HEK293T cells. (D) Endogenous interaction of RNF149 and IRF3 was detected by Co-IP in mouse peritoneal macrophages with RSV infection. (E) In vitro interaction of RNF149 and IRF3. (F) HeLa cells were transfected with Myc-RNF149 and Flag-IRF3 plasmids, stained with Myc (green) and Flag (red) antibodies, and the co-localization was detected by laser confocal microscopy. Scale bar, 25 μm. (G) Immunostaining of mouse peritoneal macrophages stained with RNF149 (green) and IRF3 (red) antibodies through laser confocal microscopy. Scale bar, 5 μm. (H) The protein level of IRF3 in WT and IRF3-KO HEK293T cells. (I) The expression of VSV was detected by RT-qPCR in WT and IRF3-KO HEK293T cells transfected with vector or Myc-RNF149 plasmids for 36 h and infected with VSV-GFP for 12 h. n=3. Expression levels were normalized to 18S mRNA expression and then to the WT-Vector sample. (J) WT and IRF3-KO HEK293T cells transfected with vector or Myc-RNF149 plasmids for 36 h were infected with VSV-GFP for 12 h and the expression of VSV-G was detected by Western blot. (K) WT and IRF3-KO HEK293T cells transfected with vector or Myc-RNF149 plasmids for 36 h were infected with VSV-GFP for 12 h and the viral titer of VSV was detected by plaque assay. n=3. (L) Western blot analysis of IRF3, IRF7 and p65 in WT and KO peritoneal macrophages infected with RSV. (M) RT-qPCR analysis of *Il6* and *Tnfa* in WT and KO peritoneal macrophages infected with RSV. n=3. Expression levels were normalized to 18S mRNA expression and then to the WT-0 sample. (I, K, M) The *P*-value was determined using a two-way ANOVA test. ****P < 0.0001, ns, not significant. Data are representative of three independent experiments.

Next, we explored the mechanism of RNF149 regulating the IFN-β signaling. We transfected Myc-RNF149 along with Flag-RIG-I, Flag-MAVS, Flag-TRAF3, Flag-TBK1, Flag-IKKε and Flag-IRF3 and performed co-immunoprecipitation assay using an anti-Myc antibody. Ectopically expressed RNF149 specifically interacted with IRF3 (Fig 5A). Additionally, we co-transfected Myc-RNF149 and Flag-IRF3 plasmids in HEK293T cells. Co-Immunoprecipitation assay was performed using an anti-Flag antibody, and the results showed that RNF149 and IRF3 still interacted with each other (Fig 5B). Furthermore, endogenous RNF149 interacted with endogenous IRF3 in both HEK293T cells and peritoneal macrophages (Fig 5C–D). The interaction in macrophages did not change with the duration of virus infection. Besides, the interaction of RNF149 and IRF3 in vitro was conducted, indicating a direct interaction between RNF149 and IRF3 (Fig 5E). Hela cells with ectopic expression of RNF149 showed colocalization with exogenous IRF3 (Fig 5F). Besides, in peritoneal macrophages, endogenous RNF149 and IRF3 also showed colocalization (Fig 5G).

To further demonstrate that RNF149 regulates viral replication through targeting IRF3, we conducted experiments in IRF3-KO HEK293T cells and the knockout of IRF3 was verified (Fig 5H). In WT HEK293T cells, RT-qPCR, Western blot analysis and viral titers showed that RNF149 overexpression enhanced VSV viral replication (Fig 5I–K). While in IRF3-KO HEK293T cells, RNF149 overexpression did not enhance VSV viral replication (Fig 5I–K). Furthermore, the expression of IRF3 was higher in *Rnf149*^*−/−*^ macrophages compared to WT macrophages and the level of IRF3 did not change with the increase of virus infection time (Fig 5L). In contrast, the protein level of IRF7 and p65 was not increased in *Rnf149*^*−/−*^ macrophages (Fig 5L). The mRNA level of *Il6* and *Tnfa* was unchanged in *Rnf149*^*−/−*^ macrophages compared to macrophages from WT mice (Fig 5M), suggesting that RNF149 specifically regulates the IRF3 pathway. In summary, these findings indicate that RNF149 regulates viral replication through IRF3.

### 5. RNF149 downregulates IRF3 protein level through the proteasome pathway

**Fig 6:**
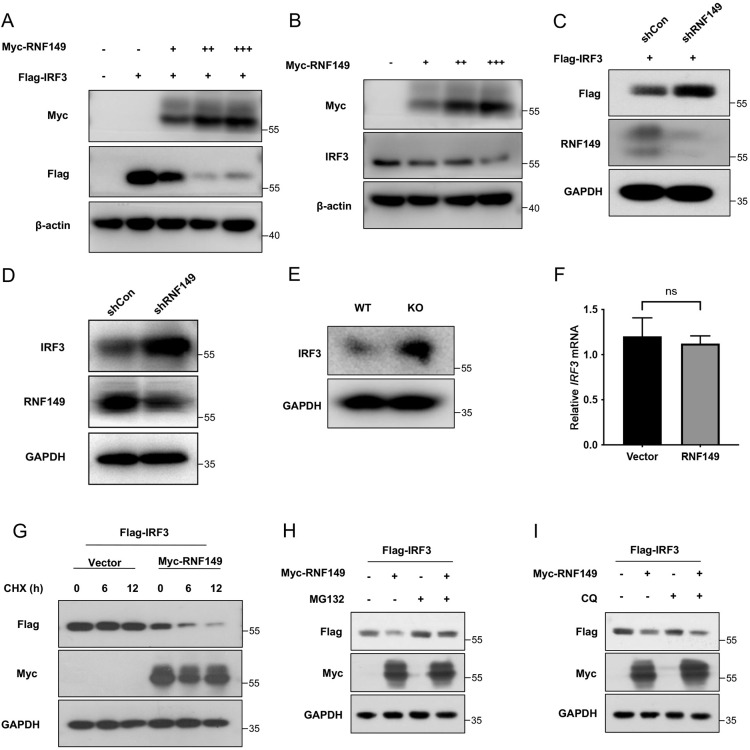
RNF149 downregulates IRF3 protein level through the proteasome pathway. (A) HEK293T cells were transfected with Flag-IRF3 and a gradient concentration of Myc-RNF149 plasmids for 48 h, and the expression of exogenous IRF3 was detected by Western blot. (B) The expression of endogenous IRF3 protein level in HEK293T cells transfected with a gradient concentration of Myc-RNF149 plasmid for 48 h. (C) HEK293T cells were transfected with shRNF149 and Flag-IRF3 plasmids for 48 h, and the expression of exogenous IRF3 was determined by Western blot. (D) The expression of endogenous IRF3 protein level in HEK293T cells transfected with shRNF149 plasmids for 48 h. (E) The expression of IRF3 in peritoneal macrophages from WT and *Rnf149*^*−/−*^ mice was detected by Western blot. (F) HEK293T cells were transfected with vector or Myc-RNF149 plasmid for 48 h, and *IRF3* mRNA was detected by RT-qPCR. n=3. Expression levels were normalized to 18S mRNA expression and then to the vector sample. (G) HEK293T cells transfected with Myc-RNF149 and Flag-IRF3 plasmids for 36 h were treated with CHX at different time points, and the protein expression of exogenous IRF3 was determined by Western blot. (H-I) HEK293T cells transfected with Myc-RNF149 and Flag-IRF3 plasmids for 36 h were treated with proteasome inhibitor MG132 or lysosome inhibitor CQ for 12 h, and the protein expression of exogenous IRF3 was determined by Western blot. (F) The *P*-value was determined using an unpaired *t*-test. ns, not significant. Data are representative of three independent experiments.

RNF149 and IRF3 exhibited interaction and co-localization, so we further explored how RNF149 regulates IRF3. In HEK293T cells, we co-transfected Myc-RNF149 and Flag-IRF3 plasmids. Gradient overexpression of RNF149 significantly reduced the expression of exogenous IRF3 (Fig 6A). Similarly, we examined the effect of RNF149 overexpression on endogenous IRF3 protein levels, and the Western blot results showed that gradient overexpression of RNF149 also notably decreased endogenous IRF3 protein expression (Fig 6B). In contrast, RNF149 knockdown upregulated the exogenous and endogenous IRF3 protein level in HEK293T cells (Fig 6C–D). Additionally, in mouse peritoneal macrophages, the IRF3 protein expression was significantly enhanced in *Rnf149*^*−/−*^ mice compared to WT mice (Fig 6E).

Next, to investigate whether the reduction of IRF3 expression by RNF149 occurs at the mRNA level or the protein level, we overexpressed RNF149 in HEK293T cells and detected the endogenous IRF3 mRNA level. RNF149 overexpression did not affect the mRNA level of IRF3 (Fig 6F), suggesting that RNF149 regulates IRF3 expression by affecting its protein level. Subsequently, to confirm whether RNF149 downregulates IRF3 expression by influencing its protein degradation, a CHX chase assay was conducted. Overexpression of RNF149 accelerated the protein degradation of Flag-IRF3 (Fig 6G). Protein degradation primarily occurs through the ubiquitin-proteasome or the lysosomal pathway [34]. To determine which pathway RNF149 mediated IRF3 degradation, we treated HEK293T cells overexpressing RNF149 and IRF3 with proteasome inhibitor MG132 or lysosomal inhibitor CQ. The results showed that the proteasome inhibitor MG132 could reverse the degradation of IRF3 induced by RNF149, while the lysosomal inhibitor CQ did not restore IRF3 expression (Fig 6H–I). In summary, these results suggest that RNF149 mediates the degradation of IRF3 through the ubiquitin-proteasome pathway.

### 6. RNF149 promotes the K27-linked and K33-linked ubiquitination of IRF3

**Fig 7:**
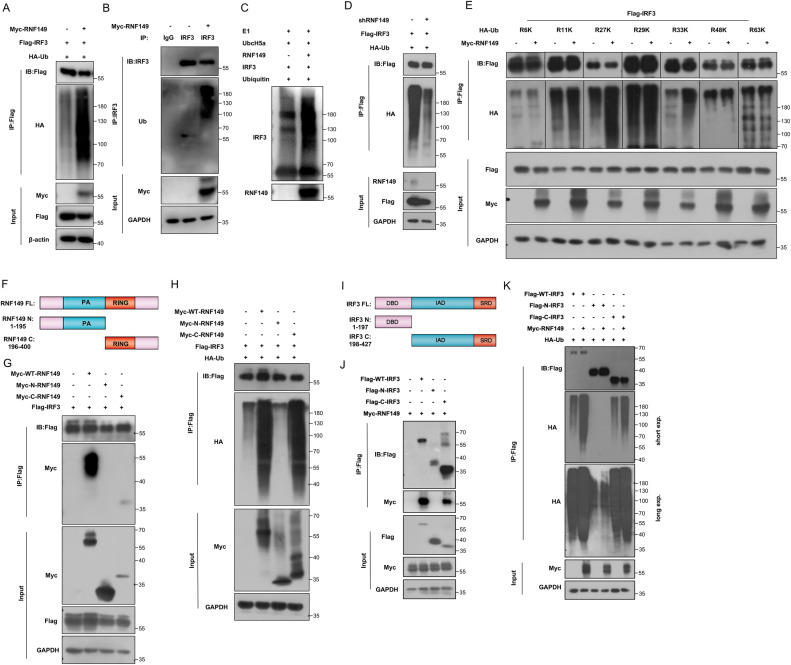
RNF149 promotes the K27-linked and K33-linked ubiquitination of IRF3. (A) HEK293T cells were transfected with Myc-RNF149, Flag-IRF3 and HA-Ub plasmids for 48 h, and the ubiquitin level of exogenous IRF3 was detected by Co-IP. (B) HEK293T cells were transfected with Myc-RNF149 plasmid for 48 h, and the ubiquitin level of endogenous IRF3 was detected by Co-IP. (C) In vitro IRF3 ubiquitination assay using RNF149 and IRF3 in the presence of E1, E2 (UbcH5A) and ubiquitin. (D) HEK293T cells were transfected with shRNF149, Flag-IRF3 and HA-Ub plasmids for 48 h, and the ubiquitin level of exogenous IRF3 was detected by Co-IP. (E) The ubiquitination level of exogenous IRF3 was detected by Co-IP in HEK293T cells co-transfected with HA-Ub-R6K, HA-Ub-R11K, HA-Ub-R27K, HA-Ub-R29K, HA-UbR33K, HA-Ub-R48K or HA-Ub-R63K along with Myc-RNF149 and Flag-IRF3 for 48 h. (F) The diagram of RNF149 protein domain. (G) HEK293T cells were co-transfected with Flag-IRF3 and Myc-WT-RNF149/ Myc-N-RNF149/ Myc-C-RNF149 and the interaction was detected by Co-IP. (H) HEK293T cells were co-transfected with Flag-IRF3, HA-Ub, and Myc-WT-RNF149/ Myc-N-RNF149/ Myc-C-RNF149 and the ubiquitination of IRF3 was determined by Co-IP. (I) The diagram of IRF3 protein domain. (J) HEK293T cells were co-transfected with Myc-RNF149 and Flag-WT-IRF3/ Flag-N-IRF3/ Flag-C-IRF3, and the interaction was detected by Co-IP. (K) HEK293T cells were co-transfected with Myc-RNF149, HA-Ub, and Flag-WT-IRF3/ Flag-N-IRF3/ Flag-C-IRF3, and the ubiquitination of WT-IRF3, N-IRF3, and C-IRF3 was detected by Co-IP. Data are representative of three independent experiments.

Based on these findings, we hypothesized that RNF149 played a role in regulating the ubiquitination of IRF3 protein. To confirm whether RNF149 can ubiquitinate IRF3, a protein ubiquitination assay was conducted. The results showed that overexpression of RNF149 significantly increased the ubiquitination of exogenous and endogenous IRF3 (Fig 7A–B). In an in vitro ubiquitination assay, RNF149 directly ubiquitinated IRF3 in the presence of E1 activating enzyme, E2 conjugating enzyme and Ubiquitin (Fig 7C). Additionally, RNF149 knockdown attenuated exogenous IRF3 ubiquitination in HEK293T cells (Fig 7D). To investigate the specific types of ubiquitination modifications of IRF3 induced by RNF149, we transfected HEK293T cells with Myc-RNF149, Flag-IRF3, and various ubiquitin mutants, including HA-R6K, HA-R11K, HA-R27K, HA-R29K, HA-R33K, HA-R48K, and HA-R63K. These mutant plasmids replace all lysine residues except for specific ones with arginine. Overexpression of RNF149 primarily induced K27 and K33-linked ubiquitination of IRF3 (Fig 7E).

RNF149 consists of two main domains: the Protease-Associated domain (PA) and the RING finger domain (RING). To identify which domain of RNF149 interacted with IRF3, we constructed plasmids with the N-terminal PA domain and the C-terminal RING domain of RNF149 (Fig 7F). The results indicated that the C-terminal RING domain of RNF149 interacted with IRF3 (Fig 7G). Additionally, we examined the effect of RNF149 domains on IRF3 ubiquitination and found that the C-terminal domain of RNF149 promoted the ubiquitination of IRF3 (Fig 7H). We have demonstrated that RNF149 interacts with IRF3 and promotes its ubiquitination. However, the specific domain of IRF3 affected by RNF149 remains unknown. IRF3 consists of three main domains: the DNA binding domain (DBD), the IRF association domain (IAD), and the signal response domain (SRD). To identify the specific domain targeted by RNF149, we constructed plasmids with the N-terminal containing the DBD domain and the C-terminal containing the IAD and SRD domains of IRF3 (Fig 7I). The results indicated that RNF149 interacted with the C-terminal of IRF3 and promoted its ubiquitination (Fig 7J–K).

Next, we explored the specific ubiquitination residue of IRF3 induced by RNF149. Based on the above findings, it is known that RNF149 interacted with the C-terminus of IRF3 and promoted its ubiquitination. Therefore, we constructed five lysine mutant plasmids of the IRF3 C-terminus, namely K313R, K315R, K360R, K366R, and K409R. The results showed that the K409 site of IRF3 was the site of RNF149-mediated K27 ubiquitination, while the K366 and K409 sites of IRF3 were the sites of RNF149-mediated K33 ubiquitination (S5A–B Fig). Moreover, we further constructed the IRF3 K366/409R double mutant plasmid, and the results showed that RNF149 significantly reduced K33 ubiquitination on the double mutant plasmid (S5C Fig). Additionally, the impact of RNF149 on the degradation of the double mutant IRF3 was examined. RNF149-mediated protein degradation disappeared after the IRF3 double mutation (S5D Fig). These results indicate that the K366 and K409 sites of IRF3 are crucial residues for RNF149-mediated ubiquitination modification and protein degradation. Taken together, RNF149 promoted the K27-linked ubiquitination of IRF3 at residue K409 and K33-linked ubiquitination of IRF3 at residue K366 and K409.

### 7. The regulatory effect of RNF149 on innate antiviral immunity depends on its ubiquitin ligase activity

**Fig 8:**
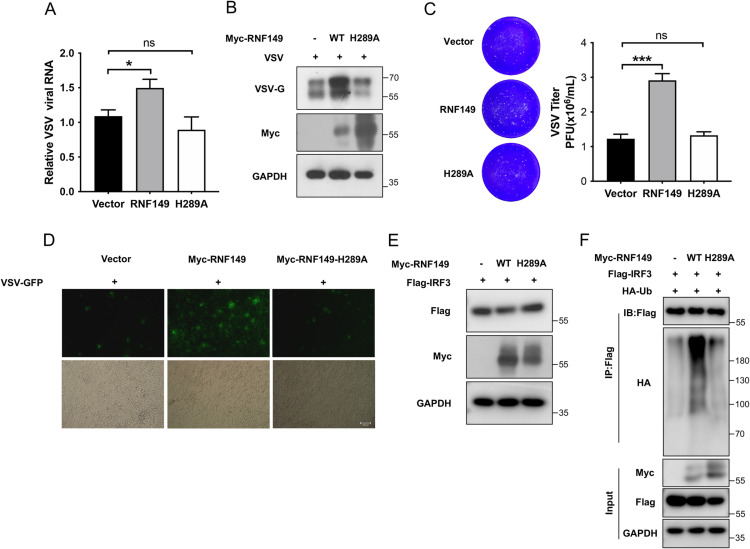
The regulatory effect of RNF149 on innate antiviral immunity is dependent on its ubiquitin ligase activity. (A) The expression of VSV was detected by RT-qPCR in HEK293T cells transfected with vector, Myc-RNF149-WT or Myc-RNF149-H289A plasmids for 36 h and infected with VSV-GFP. n=3. Expression levels were normalized to 18S mRNA expression and then to the Vector sample. (B) HEK293T cells transfected with vector, Myc-RNF149-WT or Myc-RNF149-H289A plasmids for 36 h were infected with VSV-GFP for 12 h and the expression of VSV-G was detected by Western blot. (C) HEK293T cells transfected with vector, Myc-RNF149-WT or Myc-RNF149-H289A plasmids for 36 h were infected with VSV-GFP for 12 h and the viral titer of VSV was detected by plaque assay. n=3. (D) HEK293T cells transfected with vector, Myc-RNF149-WT or Myc-RNF149-H289A plasmids for 36 h were infected with VSV-GFP for 12 h and VSV load was observed by fluorescence microscopy. Scale bar, 100 μm. (E) The protein level of IRF3 in HEK293T cells transfected with Flag-IRF3 in the presence of vector, Myc-RNF149-WT, or Myc-RNF149-H289A plasmids for 48 h. (F) The ubiquitination of IRF3 in HEK293T cells transfected with HA-Ub, Flag-IRF3 in the presence of vector, Myc-RNF149-WT, or Myc-RNF149-H289A plasmids for 48 h was determined by Co-IP. (A, C) The *P*-value was determined using an unpaired *t*-test. ns, not significant, *P <0.05, ***P < 0.001, ns, not significant. Data are representative of three independent experiments.

RNF149 mediated the ubiquitination and degradation of IRF3, thereby reducing IFN-β transcription and ultimately enhancing viral replication. We further determined whether the negative regulation of RNF149 depends on its ubiquitin ligase activity. It has been reported that the residue H289 in the RING domain of RNF149 is highly conserved and necessary for ubiquitin ligase activity, and mutation of this histidine to alanine (H289A) results in loss of ubiquitin ligase activity [27]. We constructed a Myc-RNF149-H289A plasmid and transfected it into HEK293T cells. Western blot and RT-qPCR analysis were conducted after VSV infection. RNF149-H289A could not promote VSV viral replication (Fig 8A–B). Additionally, plaque assays revealed no difference in plaque numbers between the RNF149-H289A group and the empty vector control group (Fig 8C). Fluorescence microscopy also showed similar trends in VSV fluorescence (Fig 8D). Moreover, we explored the effect of RNF149-H289A on the degradation and ubiquitination of IRF3. Western blot analysis showed that RNF149-H289A could not degrade IRF3 protein and promote its ubiquitination, while RNF149 could degrade and ubiquitinate IRF3 (Fig 8E–F). In brief, these results indicate that the regulatory effect of RNF149 on viral replication and IRF3 depends on its ubiquitin ligase activity.

## Discussion

Herein, we demonstrated a new role of E3 ubiquitin ligase RNF149 in innate antiviral immunity. Various studies have reported that RNF149 played a role in tumors, such as colorectal cancer, nasopharyngeal cancer, hepatocellular carcinoma and esophageal squamous cancer [27,28,31,35]. In addition to its role in tumors, RNF149 also has a regulatory effect in Alzheimer’s disease [29]. In this study, we first found that RNF149 also functioned on antiviral responses through degrading IRF3, thereby downregulating the IFN-β production.

Interferon-stimulated genes are induced during the interferon responses. Generally, ISGs inhibit viral infection by limiting different stages of the viral replication cycle [36]. For instance, IFITM proteins, CH25H and NCOA7 inhibit viral entry [37–39]. MX1 and MX2 hinder the transport of viral components via the nuclear pore complex [40]. IFIT proteins, ZAP and PARP12 restrict the viral protein translation [41–43]. In contrast, a small part of ISGs promotes viral infection unexpectedly. SOCS1/3, AXL, USP18 and ADAR1 negatively regulate IFN induction [44–47]. Besides, LY6E and MCOLN2 directly enhance viral infection rather than inhibit IFN signaling [48,49]. Here, we found that RNF149 could be induced by IFN-β stimulation through the STAT1 pathway. Although Chun-Kai Huang et al. showed that the activation of STAT1 induced RNF149 expression [50], we also demonstrated that as a newly described ISG, RNF149 suppressed the IFN-β production and promoted viral infection, exerting its negative effects. We found a new ISG that negatively affects innate antiviral immunity. The underlying cause of this negative regulatory phenomenon may be to trigger stronger adaptive immune responses or to prevent excessive immune responses.

Respiratory Syncytial Virus (RSV) is a major pathogen causing lower respiratory tract infection in infants. There is a complex relationship between RSV and type I interferon. RSV infection induces the expression of negative regulatory ISGs SOCS1 and SOCS3, suppressing the production of type I interferon [51,52]. Besides, some E3 ubiquitin ligases regulate RSV infection through the type I interferon signaling pathway. For instance, DTX3L promotes type I interferon production and inhibits RSV infection by promoting the ubiquitination and phosphorylation of TBK1 [53]. In addition, FBXW7 also upregulates type I interferon expression and suppresses RSV replication by enhancing the stability of RIG-I [54]. According to the research of Guo and Chen, they utilized *Ifnar1*^*−/−*^ MEF to explore the relationship between the function of their genes on virus infection and type I IFN signaling pathway [24,55]. Similarly, we found that RNF149’s effects on viral infection are linked to the type I IFN signaling pathway through MEF cells. Due to the unavailability of *Rnf149*^*−/−*^ and *Ifnar1*^*−/−*^ double-knockout mice, we conducted experiments using MEF cells, although double-knockout mice would have been more ideal for this study. Our results indicate that RNF149 suppresses type I interferon signaling and enhances RSV infection. Infants infected with RSV have lower levels of IFN-α in their respiratory secretions [56]. The susceptibility of infants to RSV is not only due to the immaturity of the immune system but may also be caused by insufficient secretion of type I interferon. Therefore, it’s essential to explore the relationship between type I interferon and RSV infection.

IRF3 is constitutively expressed in cells, providing a rapid response mechanism for cells to counter viral infections [57]. Therefore, IRF3 is essential for rapid antiviral innate immune response. Taniguchi et al. found that in *IRF3*^*−/−*^ mice, T and B cell development showed no effects, but they exhibited increased susceptibility to viral infection, accompanied by impaired induction of Type I interferons, IRF7, and IRF9 [58]. They confirmed the key role of IRF3 in the host's resistance to viral infection. Compared to IRF7, IRF3 has a longer half-life. Thus, the protein stability of IRF3 also demonstrates its primary position in the antiviral response [59]. Currently, many studies have found that E3 ubiquitin ligases of the RNF family can regulate IRF3. RNF26 negatively regulates the production of virus-induced IFN-I by promoting the autophagy-dependent degradation of IRF3 protein [60]. RNF55 directly interacts with IRF3, promoting the K48-linked polyubiquitination and proteasome-dependent degradation of IRF3, thereby downregulating TLR and RLR-mediated IFN-I induction [61]. In this research, RNF149 interacts with IRF3, regulating the ubiquitination of IRF3, thereby promoting its proteasome-dependent degradation.

The ubiquitination process is the attachment of one or more ubiquitin molecules to the target protein, which plays an essential role in regulating the function, stability, localization, and further signal transduction of the target protein. A single ubiquitin molecule can bind to the target protein to induce mono-ubiquitination. It can also form specific ubiquitin chains through its seven internal lysine residues (K6, K11, K27, K29, K33, K48, and K63), or form linear ubiquitin chains through the amino-terminal methionine (Met1) [62,63]. Currently, research on K48 and K63 linkage polyubiquitination is relatively well understood. K48 linkage polyubiquitination is associated with proteasomal degradation, while K63 linkage polyubiquitination regulates signal transduction, protein endocytosis, and enzyme activity [64]. K6-linked polyubiquitination is associated with autophagy and DNA damage response [65]. Additionally, K11, K27, and K29 polyubiquitination also play specific roles in regulating the innate immune response [19]. K33-linked polyubiquitination is the least studied. It’s associated with protein stability, protein trafficking and autophagy [65]. M1 ubiquitination plays a vital role in the activation of NF-κB and the production of interferons [66]. Several kinds of ubiquitination have been reported to lead to protein degradation, including K11, K27, K29 and K48-linked ubiquitination. But, K48-linked ubiquitination is one of the most predominant types of ubiquitin-mediated protein degradation. IRF3 is regulated by numerous E3 ubiquitin ligases, forming different types of ubiquitin chains. E3 ubiquitin ligases MID1, Ro52, RAUL, c-Cbl, TRIM26 and UBE3C induce the K48-linked polyubiquitination of IRF3 and downregulate the protein level of IRF3 [24–26,61,67,68]. RNF34 promotes the K27 and K48-linked ubiquitination of IRF3 and its degradation [69]. However, whether there is an E3 ubiquitin ligase that regulates the K33-ubiquitination of IRF3 remains unclear. Here, we showed that E3 ubiquitin ligase RNF149 promotes the K27-linked and K33-linked ubiquitination of IRF3, thereby mediating the degradation of IRF3.

Human IRF3 has 14 lysine sites, and the effect of ubiquitination at these sites is different. It has been reported that the ubiquitination of IRF3 at Lys 193 and Lys 313 or 315 could activate the RLR-induced IRF-3-mediated apoptosis pathway, potentially contributing to an antiviral response [70]. K48-linked ubiquitination at Lys70 and Lys87 of IRF3 mediated by E3 ubiquitin ligase TRIM26 promotes its degradation [67]. Besides, K48-linked ubiquitination at Lys313 of IRF3 induced by E3 ubiquitin ligase MID1 also accelerates its degradation [24]. Moreover, the K33-linked ubiquitination of IRF3 at Lys313 cleaved by DUB OTUD6B enhances its protein stability and type I IFN production [71]. In addition, the removal of ubiquitination at this site by the DUB PSMD14/POH1 prevents IRF3 from autophagy degradation [72]. These findings indicate that the ubiquitination and deubiquitination of IRF3 at different sites play important roles in regulating IRF3 function. However, the ubiquitination of IRF3 at K366 and K409 has not been reported. In this study, we found that RNF149 promotes the K27-linked ubiquitination of IRF3 at K409 and K33-linked ubiquitination of IRF3 at K366 and K409, thereby mediating the degradation of IRF3. The K409 residue can undergo two types of ubiquitination, possibly due to the formation of heterotypic ubiquitin chains. Heterotypic ubiquitin chains refer to the presence of two different types of ubiquitin chains on the same lysine residue of a substrate protein. When the ubiquitin chain extends, it forms mixed or branched ubiquitin chain modifications, and this complexity allows the chain to convey more diversified signals [73]. It’s very interesting that each lysine site of IRF3 can be modified by different E3 ubiquitin ligases and how these ubiquitin ligases control the activity, stability, and subcellular localization of IRF3, which in turn influence and keep the balance of the antiviral response.

In conclusion, we have clarified that RNF149 expression is induced upon viral infection, and RNF149 regulates the IRF3 degradation. Our study has unveiled a novel role for RNF149 in the innate immune response against viral infection, thus offering a new strategy for the treatment of RSV infection.

## Materials and methods

### Ethics statement

All animal experiments were performed by the Guide of the National Animal Care and Use Committee and the Laboratory Animal Ethical Commission of Soochow University (SYXK2015-0018).

### Mice

*Rnf149*^*−/−*^ mice were generated by Cyagen Biosciences. Genotyping of wild-type (WT) and *Rnf149*^*−/−*^ mice was performed with the following primers: forward primer 5′- TAGTGATCACAGAAGAGCTCTCACA-3′ and reverse primer 5′- AGTGAGAAAATCACCAGGAACTGT-3′ for WT mice; forward primer 5′- TAGTGATCACAGAAGAGCTCTCACA-3′ and reverse primer 5′- AGTGGAAAAGCTGGTTTAGAATCAC-3′ for *Rnf149*^*−/−*^ mice. All the mice were C57BL/6 background and kept under an SPF animal facility.

### Cells and reagents

RAW264.7, HEK293T, Vero, HeLa and Hep2 cells were purchased from the American Type Culture Collection. Mouse primary peritoneal macrophages were obtained from C57BL/6J mice (6–8 weeks) through intraperitoneal injection with 4% thioglycolate. These cells were cultured at 37°C under 5% CO_2_ in DMEM supplemented with 10% FBS, 100 U/mL penicillin and 100 µg/ml streptomycin. THP-1 cells were purchased from the American Type Culture Collection and cultured at 37°C under 5% CO_2_ in RPMI 1640 supplemented with 10% FBS, 100 U/mL penicillin and 100 µg/ml streptomycin. Recombinant mouse IFN-β was purchased from R&D Systems.

### Sequences, plasmids and transfection

Myc-WT-RNF149, Myc-N-RNF149, Myc-C-RNF149, Myc-RNF149-H289A, Flag-N-IRF3, Flag-C-IRF3, Flag-IRF3-K313R, Flag-IRF3-K315R, Flag-IRF3-K360R, Flag-IRF3-K366R, Flag-IRF3-K409R and Flag-IRF3-K366/K409R were purchased from Youbio Biological Technology Co., Ltd. HA-ubiquitin (HA-Ub), HA-R6K, HA-R11K, HA-R27K, HA-R29K, HA-R33K, HA-R48K and HA-R63K were gifts from Dr. Hui Zheng (Soochow University, China). RNF149 promoter reporter plasmid was obtained from GenScript. The sh-human-RNF149 was generated with following primers: forward primer 5′- TGCCCATGTCTCACGCGGGAATTCAAGAGATTCCCGCGTGAGACATGGGTTTTTTC-3′ and reverse primer 5′- TCGAGAAAAAACCCATGTCTCACGCGGGAATCTCTTGAATTCCCGCGTGAGACATGGGCA. Human and mouse RNF149 siRNAs were purchased from Genepharma, and STAT1 siRNA and STAT2 siRNA were obtained from Santa Cruz Biotechnology. The sequences of human RNF149 siRNA were sense 5′- CCCAUGUCUCACGCGGGAATT-3′ and antisense 5′- UUCCCGCGUGAGACAUGGGTT-3′. The sequences of mouse RNF149 siRNA were sense 5′- GGAGACUAAGAAGGUUAUUTT-3′ and antisense 5′- AAUAACCUUCUUAGUCUCCTT-3′. For transient transfection of plasmids and siRNA into HEK293 cells, a D-Portal transfection reagent was used according to the manufacturer's instructions. For transient transfection of siRNA into RAW264.7 cells, CALNP RNAi in vitro transfection reagent was used according to the manufacturer's instructions.

### Virus and viral infection

RSV (L19 strain) was a gift from Dr. Chunsheng Dong (Soochow University, China). VSV-GFP, SeV and HSV-1 were gifts from Dr. Hui Zheng (Soochow University, China). VSV was a gift from Dr. Fangfang Zhou (Soochow University, China). Cells were infected with RSV (MOI=10), VSV (MOI=0.1) or SeV (100 hemagglutination units [HAU]/mL) in 2% medium for 2 h. Then the medium was removed, and cells were cultured in the fresh 2% FBS medium for the indicated time. The cells were determined by RT-qPCR or Western blot. For in vivo viral infection, WT or *Rnf149*^*−/−*^ mice (6-8 weeks) were infected with 1×10^9^ PFU/mouse of RSV or PBS through nasal instillation. IFN-β in the sera was measured by ELISA. The mRNA level of RSV in the lungs was detected by RT-qPCR. Lungs from control and RSV-infected mice were fixed in 4% paraformaldehyde, embedded into paraffin, sectioned, stained with hematoxylin-eosin solution and examined by microscopy for histological changes. WT or *Rnf149*^*−/−*^ mice (6-8 weeks) were infected with 4×10^8^ PFU/mouse of VSV or PBS through intraperitoneal injection. VSV viral loads in the lungs, kidneys, spleens and livers were determined by RT-qPCR or Western blot. For the survival experiments, mice were monitored for survival after VSV infection.

### RNA isolation and quantification

Total RNA was extracted according to the procedure of TRIzol reagent (Vazyme) and reversely transcribed into cDNA using Hifair Ⅲ 1st Strand cDNA Synthesis SuperMix for qPCR (Yeasen) referring to the instruction. RT-qPCR was performed using Hieff qPCR SYBR Green Master Mix (Low Rox Plus) (Yeasen) and detected by QuantStudio 1 (Thermo Fisher Scientific). The relative expression of the target genes was normalized to 18S mRNA. The primers are listed in S1 Table.

### Western blot and co-immunoprecipitation

Cells or tissues were lysed with NP-40 or RIPA Lysis Buffer (Beyotime) with PMSF (Beyotime). After centrifugation at 12,000 rpm for 20 min, whole-cell lysates were used for immunoblotting or immunoprecipitation. For co-immunoprecipitation analysis, lysates were incubated with Protein G-Agarose beads (Roche) and corresponding antibodies. After 5 h of incubation, beads were washed with twice NP-40 washing buffer and once with high-salt washing buffer. Immunoprecipitates were eluted by SDS-PAGE Loading Buffer and boiled for 10 min. For Western blot analysis, immunoprecipitates or whole-cell lysates were subjected to SDS-PAGE, transferred onto PVDF membranes and then blotted with indicated antibodies. The antibodies used were as follows: RNF149 (NBP2-93343, Novus biologicals), STAT1 (14994S, Cell Signaling Technology), STAT2 (A14995, Abclonal), VSV-G (ab1874, Abcam), IRF3 (11312-1-AP, Proteintech), IRF3 (sc-9082, Santa Cruz), HA (T0008, Affinity Biosciences), Myc (AF6055, Affinity Biosciences), Flag (T0003, Affinity Biosciences), Flag (T0053, Affinity Biosciences), Ubiquitin (Ub) (sc-8017, Santa Cruz), β-actin (T0022, Affinity Biosciences), GAPDH (60004-1-Ig, Proteintech), MX1 (13750-1-AP, Proteintech) and CXCL10 (ab9938, Abcam).

### Immunofluorescence and confocal microscopy assay

HeLa cells and peritoneal macrophages cultured on a chamber slide were fixed with 4% paraformaldehyde for 30 min at room temperature, permeabilized with 0.2% Triton X-100 for 5 min, and blocked with 3% BSA for 1 h. Then, the cells were incubated with primary antibodies overnight and further stained with suitable Goat Anti-Rabbit IgG-AF488–conjugated secondary antibodies (4050-30, Southern Biotech) or Goat anti-Mouse IgG AF647–conjugated secondary antibodies (A21235, Invitrogen).

### Luciferase reporter assay

HEK293 cells were co-transfected with RNF149 promoter reporter plasmid and pRL-TK, together with the STAT1 expression or control vector plasmid. Luciferase activity was detected by the Dual-Luciferase Reporter Assay system according to the manufacturer's instructions (DD1205-01, Vazyme). The results were normalized by the division of firefly luciferase activity by Renilla luciferase.

### Statistical analysis

Statistical analysis was determined by an unpaired two-tailed *t*-test or a two-way ANOVA test. The survival curve was determined by a Log-rank (Mantel-Cox) test. Data were presented as mean ± SD (standard derivation). *P* values < 0.05 were considered statistically significant.

## Supporting information

S1 Fig RNF149 is an interferon-stimulated gene. (A) RT-qPCR analysis of *Rnf149* expression in RAW264.7 cells treated with IFN-β for 12 h at different concentrations. n=3. Expression levels were normalized to 18S mRNA expression and then to the 0 sample. (B) Western blot analysis of RNF149 expression in RAW264.7 cells treated with IFN-β at different concentrations. (C) RAW264.7 cells were blocked by IFNAR1 antibody for 2 h and infected with RSV for 12 h. The expression of *Rnf149* was detected by RT-qPCR. n=3. Expression levels were normalized to 18S mRNA expression and then to the IFNAR1-Ab(-)-RSV(-) sample. (D) The binding of STAT1 to the RNF149 promoter region in HeLa cells was detected by CHIP-seq in the ENCODE database. (E) Luciferase activity in HEK293T cells transfected with Flag-STAT1, pRL-TK and RNF149 promoter reporter plasmids for 24 h. n=5. (F) RT-qPCR analysis of *Rnf149*, *Stat1* and *Stat2* in RAW264.7 cells transfected with control siRNA, siSTAT1 or siSTAT2 for 36 h and then infected with RSV for 12 h. n=3. Expression levels were normalized to 18S mRNA expression and then to the NC-RSV(-) sample. (G) Western blot analysis of RNF149, STAT1 and STAT2 in RAW264.7 cells transfected with control siRNA, siSTAT1 or siSTAT2 for 36 h and then infected with RSV for 12 h. (A, C, E, F) The *P*-value was determined using an unpaired *t*-test. **P < 0.01, ***P < 0.001, ****P < 0.0001. Data are representative of three independent experiments.
(TIF)

S2 Fig Overexpression of RNF149 promotes viral replication. (A) HEK293T cells were transfected with vector or Myc-RNF149 for 36 h and infected with SeV, VSV, RSV or HSV-1 for 12 h, and the expression of RNF149 and virus RNA was detected by RT-qPCR. n=3. Expression levels were normalized to 18S mRNA expression and then to the vector sample. (B) HEK293T cells were transfected with vector or Myc-RNF149 for 36 h and infected with VSV-GFP for 12 h. The fluorescence of VSV was detected by fluorescence microscopy. Scale bar, 200μm. (C) HEK293T cells were transfected with vector or Myc-RNF149 for 36 h and infected with RSV-mCherry for 12 h. The fluorescence of RSV was detected by fluorescence microscopy. Scale bar, 200 μm. (D) HEK293T cells were transfected with vector or Myc-RNF149 for 36 h and infected with VSV-GFP for 12 h, and the expression of VSV-G was detected by Western blot. (E) HEK293T cells were transfected with vector or Myc-RNF149 for 36 h and infected with VSV-GFP for 12 h. The VSV viral load was detected by plaque assay. n=3. (A, E) The *P*-value was determined using an unpaired *t*-test. **P < 0.01, ***P < 0.001, ****P < 0.0001. Data are representative of three independent experiments.
(TIF)

S3 Fig Knocking down of RNF149 promotes the production of IFN-β and interferon-stimulated genes. (A) The expression of *IFNB* mRNA in HEK293T cells transfected with vector or Myc-RNF149 for 36 h and infected with SeV, VSV, RSV or HSV-1 for 12 h was detected by RT-qPCR. n=3. Expression levels were normalized to 18S mRNA expression and then to the Vector-PBS sample. (B) The expression of *IFNB* mRNA in HEK293T cells transfected with shCon or shRNF149 for 36 h and infected with SeV, VSV, RSV or HSV-1 for 12 h was detected by RT-qPCR. n=3. Expression levels were normalized to 18S mRNA expression and then to the shCON-PBS sample. (C) The expression of *IFIT1*, *ISG15,* and *ISG54* mRNA in HEK293T cells transfected with shCon or shRNF149 for 36 h and infected with SeV for 12 h was detected by qPCR. n=3. Expression levels were normalized to 18S mRNA expression and then to the shCON-PBS sample. (A-C) The *P*-value was determined using a two-way ANOVA test. ***P < 0.001, ****P < 0.0001. Data are representative of three independent experiments.
(TIF)

S4 Fig *Rnf149*^*−/−*^ mice model is constructed. (A) Construction strategy and DNA sequencing identification of *Rnf149*^*−/−*^ mice. (B) Mouse tail DNA PCR identification of *Rnf149*^*−/−*^ mice. WT: wild-type mice, KO: *Rnf149*^*−/−*^ mice and HET: *Rnf149*^*+/−*^ mice. (C) RT-qPCR was used to detect *Rnf149* mRNA levels in macrophages of *Rnf149*^*−/−*^ mice. n=3. (D) Western blot analysis of RNF149 protein levels in *Rnf149*^*−/−*^ mouse macrophages. (C) The *P*-value was determined using an unpaired *t*-test. **P < 0.01. Data are representative of three independent experiments (B-D).
(TIF)

S5 Fig RNF149 promotes the polyubiquitination of IRF3 at Lys366 and Lys409. (A) HEK293T cells were transfected with Flag-IRF3-WT, Flag-IRF3-K313R, Flag-IRF3- K315R, Flag-IRF3-K360R, FlagIRF3-K366R, Flag-IRF3-K409R along with Myc-RNF149 and HA-Ub-R27K for 48 h and the ubiquitination of IRF3 was detected by co-immunoprecipitation. (B) HEK293T cells were transfected with Flag-IRF3-WT, Flag-IRF3-K313R, Flag-IRF3-K315R, Flag-IRF3-K360R, FlagIRF3-K366R, Flag-IRF3- K409R along with Myc-RNF149 and HA-Ub-R33K for 48 h and the ubiquitination of IRF3 was detected by co-immunoprecipitation. (C) HEK293T cells were transfected with HA-Ub-R33K and Flag-IRF3-WT/ Flag-IRF3-K366/409R in the presence of vector or Myc-RNF149 for 48 h and the ubiquitination of IRF3 was detected by co-immunoprecipitation. (D) The expression of IRF3 was detected by Western blot in HEK293T transfected with Flag-IRF3-WT or Flag-IRF3-K366/409R along with Myc-RNF149 for 48 h, and quantification of band intensity of Flag in the blot, presented relative to GAPDH. n=3. The *P*-value was determined using an unpaired *t*-test. ****P < 0.0001, ns, not significant. Data are representative of three independent experiments.
(TIF)

S1 Table Sequences of primers used in RT-qPCR. (DOCX)

S1 Data The underlying numerical data and statistical analysis in this study. (XLSX)
