# Supplementary figures and images for: RNF149 modulates the type I IFN innate antiviral immune responses through degrading IRF3
Source: PLoS Pathog. 2025 Apr 17;21(4):e1013051. doi: 10.1371/journal.ppat.1013051 (PMC12005527; doi:10.1371/journal.ppat.1013051)

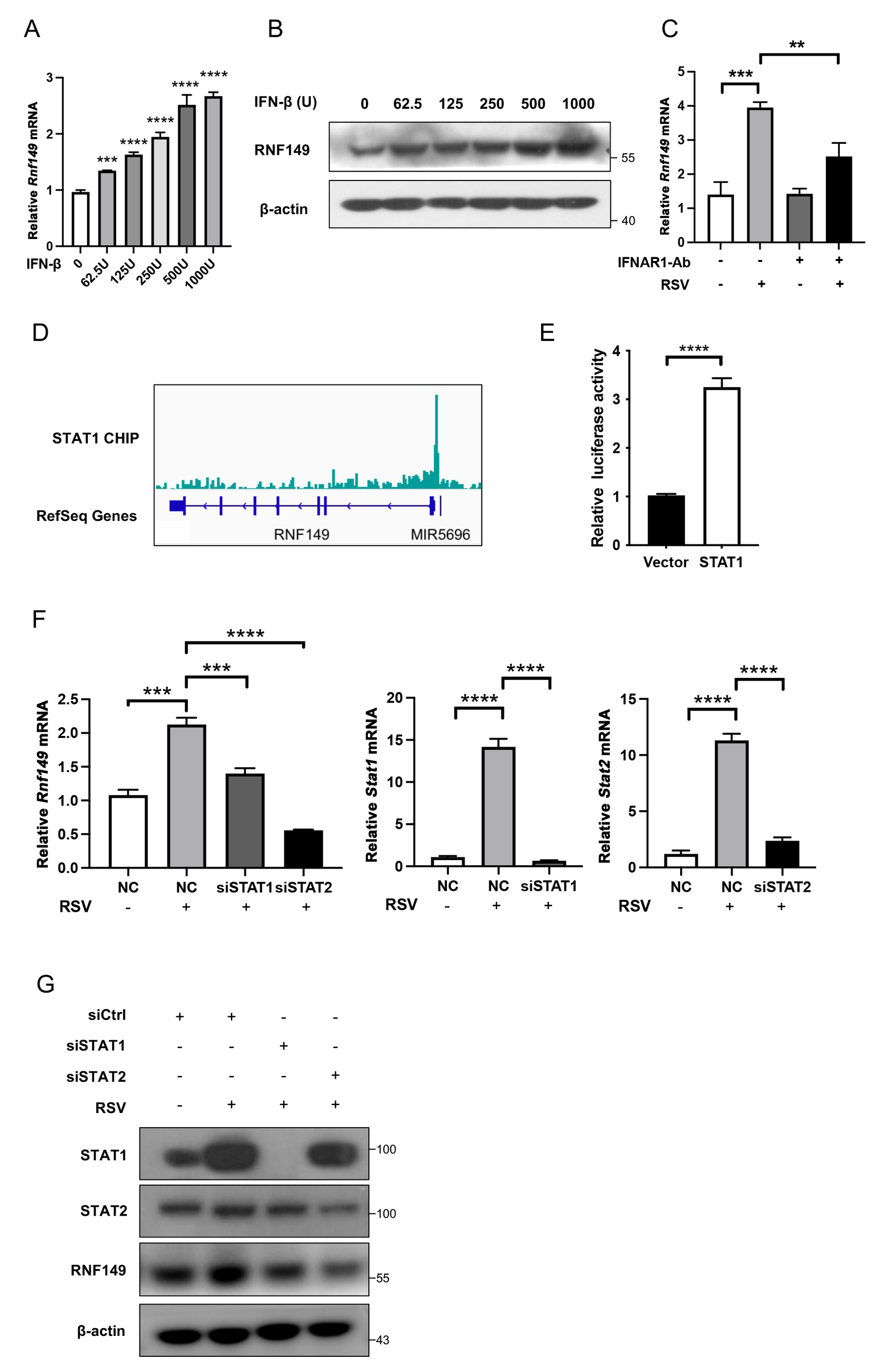

Supplement: S1 Fig — (A) RT-qPCR analysis of Rnf149 expression in RAW264.7 cells treated with IFN-β for 12 h at different concentrations. n=3. Expression levels were normalized to 18S mRNA expression and then to the 0 sample. (B) Western blot analysis of RNF149 expression in RAW264.7 cells treated with IFN-β at different concentrations. (C) RAW264.7 cells were blocked by IFNAR1 antibody for 2 h and infected with RSV for 12 h. The expression of Rnf149 was detected by RT-qPCR. n=3. Expression levels were normalized to 18S mRNA expression and then to the IFNAR1-Ab(-)-RSV(-) sample. (D) The binding of STAT1 to the RNF149 promoter region in HeLa cells was detected by CHIP-seq in the ENCODE database. (E) Luciferase activity in HEK293T cells transfected with Flag-STAT1, pRL-TK and RNF149 promoter reporter plasmids for 24 h. n=5. (F) RT-qPCR analysis of Rnf149, Stat1 and Stat2 in RAW264.7 cells transfected with control siRNA, siSTAT1 or siSTAT2 for 36 h and then infected with RSV for 12 h. n=3. Expression levels were normalized to 18S mRNA expression and then to the NC-RSV(-) sample. (G) Western blot analysis of RNF149, STAT1 and STAT2 in RAW264.7 cells transfected with control siRNA, siSTAT1 or siSTAT2 for 36 h and then infected with RSV for 12 h. (A, C, E, F) The P-value was determined using an unpaired t-test. **P < 0.01, ***P < 0.001, ****P < 0.0001. Data are representative of three independent experiments. (TIF) [file ppat.1013051.s001.tif]

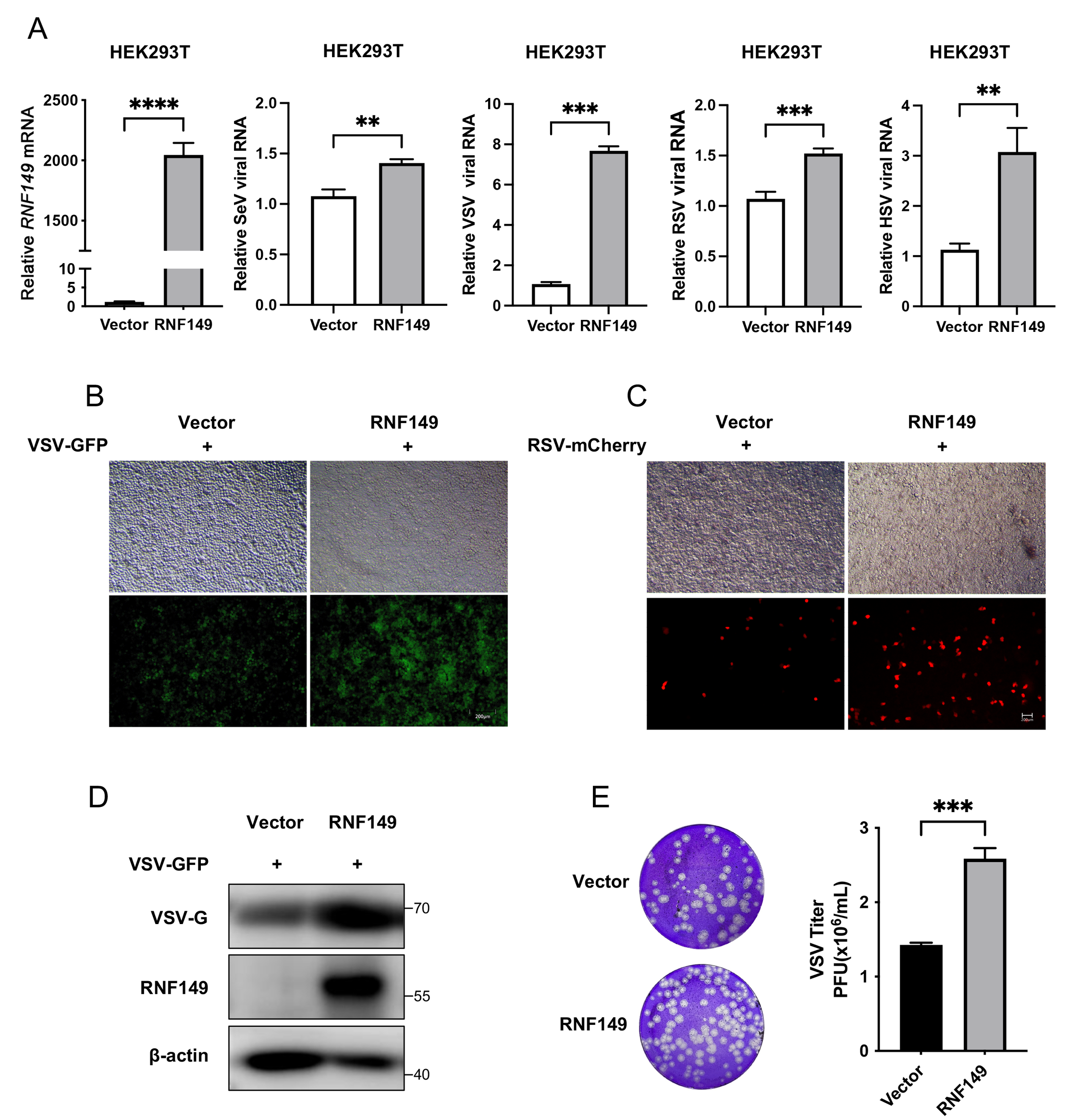

Supplement: S2 Fig — (A) HEK293T cells were transfected with vector or Myc-RNF149 for 36 h and infected with SeV, VSV, RSV or HSV-1 for 12 h, and the expression of RNF149 and virus RNA was detected by RT-qPCR. n=3. Expression levels were normalized to 18S mRNA expression and then to the vector sample. (B) HEK293T cells were transfected with vector or Myc-RNF149 for 36 h and infected with VSV-GFP for 12 h. The fluorescence of VSV was detected by fluorescence microscopy. Scale bar, 200μm. (C) HEK293T cells were transfected with vector or Myc-RNF149 for 36 h and infected with RSV-mCherry for 12 h. The fluorescence of RSV was detected by fluorescence microscopy. Scale bar, 200 μm. (D) HEK293T cells were transfected with vector or Myc-RNF149 for 36 h and infected with VSV-GFP for 12 h, and the expression of VSV-G was detected by Western blot. (E) HEK293T cells were transfected with vector or Myc-RNF149 for 36 h and infected with VSV-GFP for 12 h. The VSV viral load was detected by plaque assay. n=3. (A, E) The P-value was determined using an unpaired t-test. **P < 0.01, ***P < 0.001, ****P < 0.0001. Data are representative of three independent experiments. (TIF) [file ppat.1013051.s002.tif]

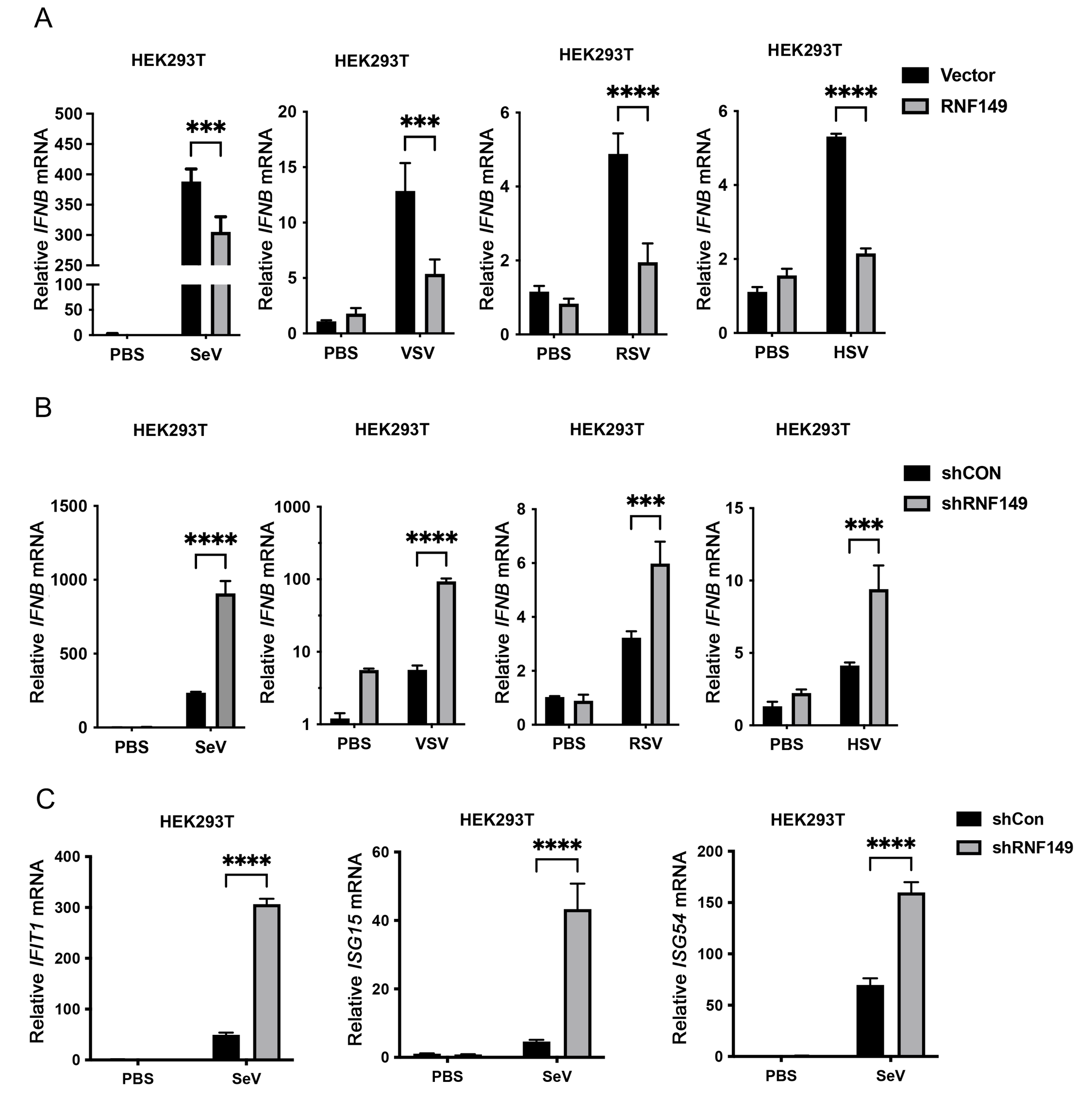

Supplement: S3 Fig — (A) The expression of IFNB mRNA in HEK293T cells transfected with vector or Myc-RNF149 for 36 h and infected with SeV, VSV, RSV or HSV-1 for 12 h was detected by RT-qPCR. n=3. Expression levels were normalized to 18S mRNA expression and then to the Vector-PBS sample. (B) The expression of IFNB mRNA in HEK293T cells transfected with shCon or shRNF149 for 36 h and infected with SeV, VSV, RSV or HSV-1 for 12 h was detected by RT-qPCR. n=3. Expression levels were normalized to 18S mRNA expression and then to the shCON-PBS sample. (C) The expression of IFIT1, ISG15, and ISG54 mRNA in HEK293T cells transfected with shCon or shRNF149 for 36 h and infected with SeV for 12 h was detected by qPCR. n=3. Expression levels were normalized to 18S mRNA expression and then to the shCON-PBS sample. (A-C) The P-value was determined using a two-way ANOVA test. ***P < 0.001, ****P < 0.0001. Data are representative of three independent experiments. (TIF) [file ppat.1013051.s003.tif]

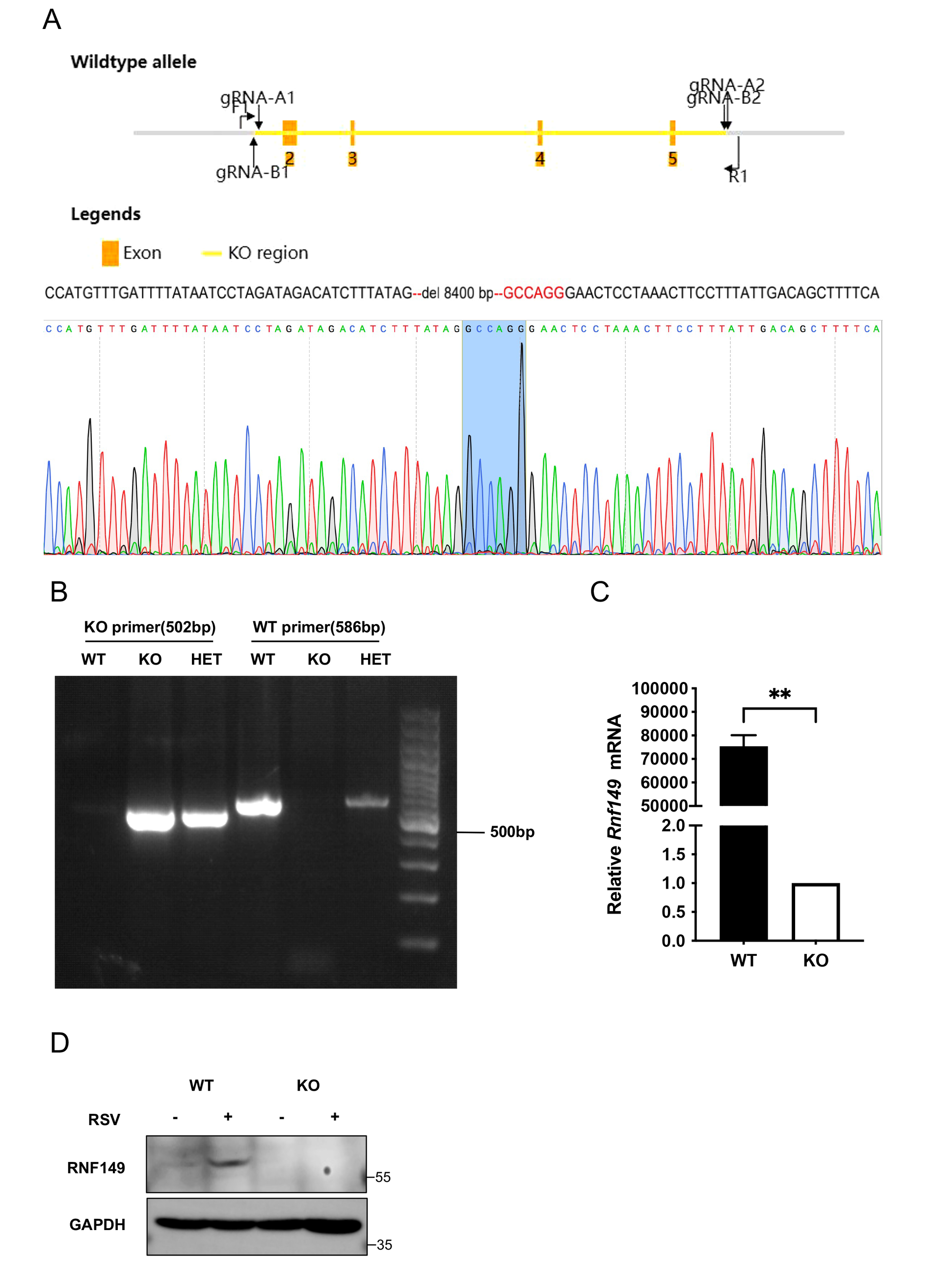

Supplement: S4 Fig — (A) Construction strategy and DNA sequencing identification of Rnf149−/− mice. (B) Mouse tail DNA PCR identification of Rnf149−/− mice. WT: wild-type mice, KO: Rnf149−/− mice and HET: Rnf149+/− mice. (C) RT-qPCR was used to detect Rnf149 mRNA levels in macrophages of Rnf149−/− mice. n=3. (D) Western blot analysis of RNF149 protein levels in Rnf149−/− mouse macrophages. (C) The P-value was determined using an unpaired t-test. **P < 0.01. Data are representative of three independent experiments (B-D). (TIF) [file ppat.1013051.s004.tif]

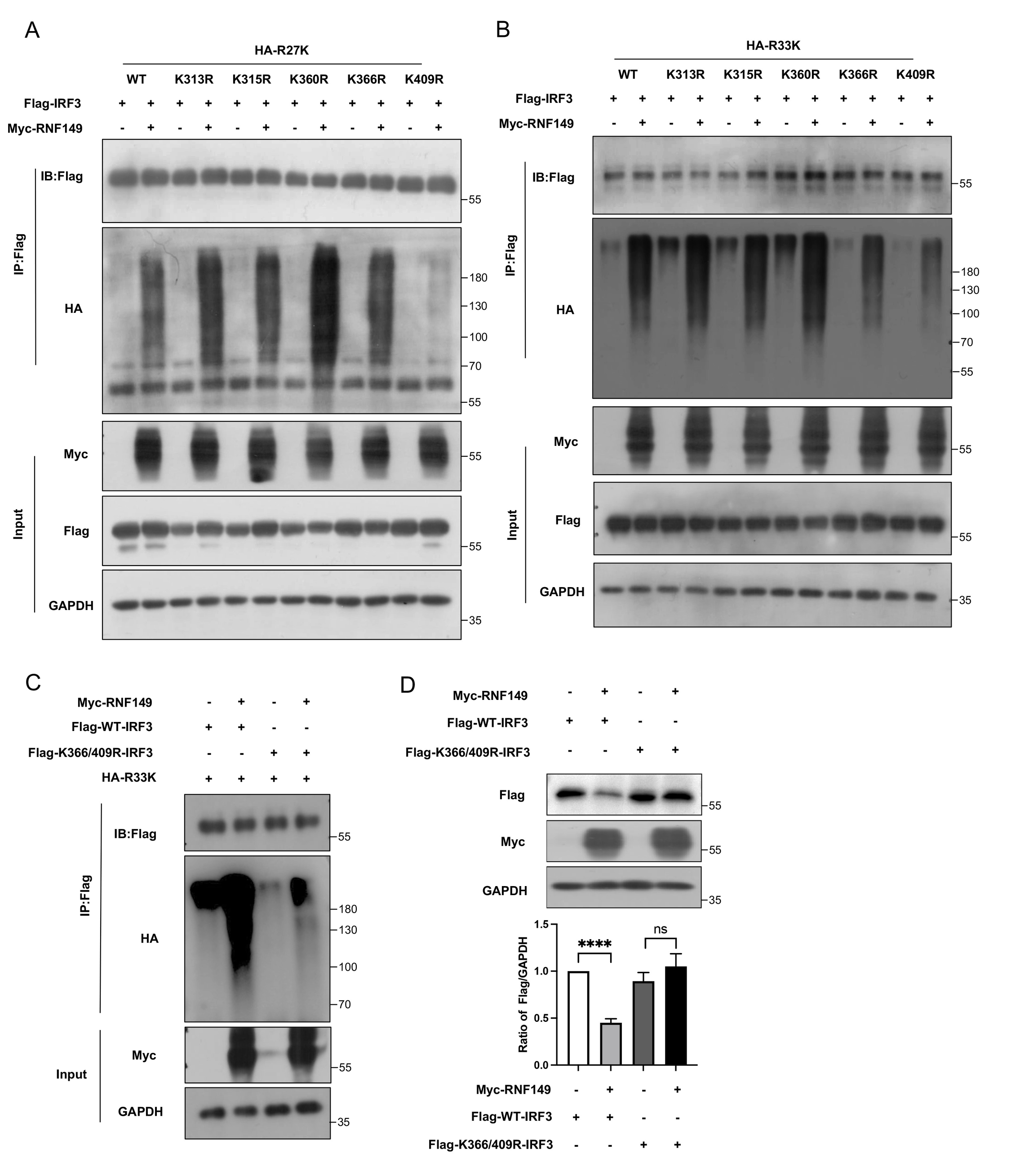

Supplement: S5 Fig — (A) HEK293T cells were transfected with Flag-IRF3-WT, Flag-IRF3-K313R, Flag-IRF3- K315R, Flag-IRF3-K360R, FlagIRF3-K366R, Flag-IRF3-K409R along with Myc-RNF149 and HA-Ub-R27K for 48 h and the ubiquitination of IRF3 was detected by co-immunoprecipitation. (B) HEK293T cells were transfected with Flag-IRF3-WT, Flag-IRF3-K313R, Flag-IRF3-K315R, Flag-IRF3-K360R, FlagIRF3-K366R, Flag-IRF3- K409R along with Myc-RNF149 and HA-Ub-R33K for 48 h and the ubiquitination of IRF3 was detected by co-immunoprecipitation. (C) HEK293T cells were transfected with HA-Ub-R33K and Flag-IRF3-WT/ Flag-IRF3-K366/409R in the presence of vector or Myc-RNF149 for 48 h and the ubiquitination of IRF3 was detected by co-immunoprecipitation. (D) The expression of IRF3 was detected by Western blot in HEK293T transfected with Flag-IRF3-WT or Flag-IRF3-K366/409R along with Myc-RNF149 for 48 h, and quantification of band intensity of Flag in the blot, presented relative to GAPDH. n=3. The P-value was determined using an unpaired t-test. ****P < 0.0001, ns, not significant. Data are representative of three independent experiments. (TIF) [file ppat.1013051.s005.tif]
