# Supplementary material for: RNF149 modulates the type I IFN innate antiviral immune responses through degrading IRF3
Source: PLoS Pathog. 2025 Apr 17;21(4):e1013051. doi: 10.1371/journal.ppat.1013051 (PMC12005527; doi:10.1371/journal.ppat.1013051)
Supplement: S1 Table — (DOCX) [file ppat.1013051.s006.docx]

# Supplementary Table

**Table S1. Sequences of primers used in RT-qPCR**

| Gene | Forward primer（5′-3′） | Reverse primer（5′-3′） |
| --- | --- | --- |
| 18S | CGGCTACCACATCCAAGGAA | GCTGGAATTACCGCGGCT |
| Mus-*Rnf149* | ACTGTGAAGCATGGCGAGAA | CGTTCGGTGATCCAAAAGCC |
| Human-*RNF149* | GCCTGCTCCAGAATCTCCTC | TGTGGCTCAGATTCAGCAGG |
| Mus-*Stat1* | GCCTCTCATTGTCACCGAAGAAC | TGGCTGACGTTGGAGATCACCA |
| Mus-*Stat2* | CTGAAGGACGAACAGGATGTC | CAGGGTGGTTAATCGGCCAA |
| SeV | GATGACGATGCCGCAGCAGTAG | CCTCCGATGTCAGTTGGTTCACTC |
| VSV | ACGGCGTACTTCCAGATGG | CTCGGTTCAAGATCCAGGT |
| HSV | CGCATCAAGACCACCTCCTC | AGCTTGCGGGCCTCGTT |
| RSV | GAATTGCAGTTGCTCATGCAA | TGGCGATTGCAGATCCAACA |
| Mus-*Ifnb* | ATGAGTGGTGGTTGCAGGC | TGACCTTTCAAATGCAGTAGATTCA |
| Mus-*Cxcl10* | CCAAGTGCTGCCGTCATTTT | GATAGGCTCGCAGGGATGAT |
| Mus-*Mx1* | CTGAGATGACCCAGCACCTG | GCTGCACTTACTGGTGTCCT |
| Human-*IFNB* | CATTACCTGAAGGCCAAGGA | CAGCATCTGCTGGTTGAAGA |
| Human-*IFIT1* | CACAAGCCATTTTCTTTGCT | ACTTGGCTGCATATCGAAAG |
| Human-*ISG15* | GGGACCTGACGGTGAAGATG | CGCCGATCTTCTGGGTGAT |
| Human-*ISG54* | CACCTCTGGACTGGCAATAGC | GTCAGGATTCAGCCGAATGG |
| Human-*IRF3* | GATGCACAGCAGGAGGATTT | GTCCTCTGCTAAACGCAACC |
